# Thousands of Qatari genomes inform human migration history and improve imputation of Arab haplotypes

**DOI:** 10.1038/s41467-021-25287-y

**Published:** 2021-10-12

**Authors:** Rozaimi Mohamad Razali, Juan Rodriguez-Flores, Mohammadmersad Ghorbani, Haroon Naeem, Waleed Aamer, Elbay Aliyev, Ali Jubran, Qatar Genome Program Research Consortium, Said I. Ismail, Wadha Al-Muftah, Radja Badji, Hamdi Mbarek, Dima Darwish, Tasnim Fadl, Heba Yasin, Maryem Ennaifar, Rania Abdellatif, Fatima Alkuwari, Muhammad Alvi, Yasser Al-Sarraj, Chadi Saad, Asmaa Althani, Eleni Fethnou, Fatima Qafoud, Eiman Alkhayat, Nahla Afifi, Sara Tomei, Wei Liu, Stephan Lorenz, Najeeb Syed, Hakeem Almabrazi, Fazulur Rehaman Vempalli, Ramzi Temanni, Tariq Abu Saqri, Mohammedhusen Khatib, Mehshad Hamza, Tariq Abu Zaid, Ahmed El Khouly, Tushar Pathare, Shafeeq Poolat, Rashid Al-Ali, Omar Albagha, Souhaila Al-Khodor, Mashael Alshafai, Ramin Badii, Lotfi Chouchane, Xavier Estivill, Khalid A. Fakhro, Younes Mokrab, Jithesh V. Puthen, Karsten Suhre, Zohreh Tatari, Andrew G. Clark, Khalid A. Fakhro, Younes Mokrab

**Affiliations:** 1 Department of Human Genetics, Sidra Medicine, Doha, Qatar; 2 Department of Genetic Medicine, Weill Cornell Medicine, New York, NY USA; 3 Department of Molecular Biology and Genetics, Cornell University, New York, NY USA; 4 Weill Cornell Medicine-Qatar, Doha, Qatar; 5 College of Health and Life Sciences, Hamad Bin Khalifa University, Doha, Qatar; 6 Qatar Genome Program, Qatar Foundation Research Development and Innovation, Qatar Foundation, Doha, Qatar; 7 Qatar Biobank for Medical Research, Qatar Foundation, Doha, Qatar; 8 Integrated Genomics Services, Sidra Medicine, Doha, Qatar; 9 Applied Bioinformatics Core, Sidra Medicine, Doha, Qatar; 10 Biomedical Informatics, Sidra Medicine, Doha, Qatar; 11 Microbiome and Biomarkers Discovery lab, Sidra Medicine, Doha, Qatar; 12 College of Health Sciences, Qatar University, Doha, Qatar; 13 Molecular Genetics Lab, Hamad Medical Corporation, Doha, Qatar; 14 Department of Genetic Medicine, Microbiology and Immunology, Weill Cornell Medicine-Qatar, Doha, Qatar; 15 Research Branch, Sidra Medicine, Doha, Qatar; 16 Genomic Medicine Lab, Sidra Medicine, Doha, Qatar; 17 Medical and Population Genomics Lab, Sidra Medicine, Doha, Qatar; 18 Bioinformatics Core, Weill Cornell Medicine-Qatar, Education City, Doha, Qatar; 19 Department of Biophysics and Physiology, Weill Cornell Medicine, New York, NY USA; 20 Clinical Research Centre, Sidra Medicine, Doha, Qatar

**Keywords:** Evolutionary biology, Genomics, Population genetics

## Abstract

Arab populations are largely understudied, notably their genetic structure and history. Here we present an in-depth analysis of 6,218 whole genomes from Qatar, revealing extensive diversity as well as genetic ancestries representing the main founding Arab genealogical lineages of Qahtanite (Peninsular Arabs) and Adnanite (General Arabs and West Eurasian Arabs). We find that Peninsular Arabs are the closest relatives of ancient hunter-gatherers and Neolithic farmers from the Levant, and that founder Arab populations experienced multiple splitting events 12–20 kya, consistent with the aridification of Arabia and farming in the Levant, giving rise to settler and nomadic communities. In terms of recent genetic flow, we show that these ancestries contributed significantly to European, South Asian as well as South American populations, likely as a result of Islamic expansion over the past 1400 years. Notably, we characterize a large cohort of men with the ChrY J1a2b haplogroup (n = 1,491), identifying 29 unique sub-haplogroups. Finally, we leverage genotype novelty to build a reference panel of 12,432 haplotypes, demonstrating improved genotype imputation for both rare and common alleles in Arabs and the wider Middle East.

## Abstract (web summary)

Arab populations are relatively understudied, especially their genetic architecture and historical relationship with early founders of the ancient Near East. Here, the authors examine 6,218 Qatari whole genomes, revealing insights on migration, population history and genetic structure of populations across the Middle Eastern region.

## Introduction

The Middle East constitutes a historic intersection of human civilization and migration^1,2^. The lack of sufficient whole-genome data from this region has limited large-scale analysis of its diversity and disease risk in Arab populations. Previous studies using hundreds of subjects gave insight to the history of these populations and impact of high consanguinity and tribalism on the prevalence of genetic diseases^3–8^. Indigenous Arabs were shown to form an outgroup to non-Africans and have little Neanderthal ancestry, suggesting their migration out of Africa instead of back to Africa^9^. Evidence from ancient human DNA suggested that the earliest populations of the Near East (which overlaps with contemporary Middle East), were derived from a Basal Eurasian lineage with minor Neanderthal admixture, and populated Anatolia, Levant and Iran prior to spreading to Europe, East Africa and Eurasian Stepp, respectively^10^. Iranian farmers arrived in Western Mediterranean by the Bronze age, during which this region was populated by a mix of Iranian, Steppe and North African ancestries^11^. The exact relationship between modern Arabs and the early founders of the ancient Near East remains unclear.

**Fig. 1:**
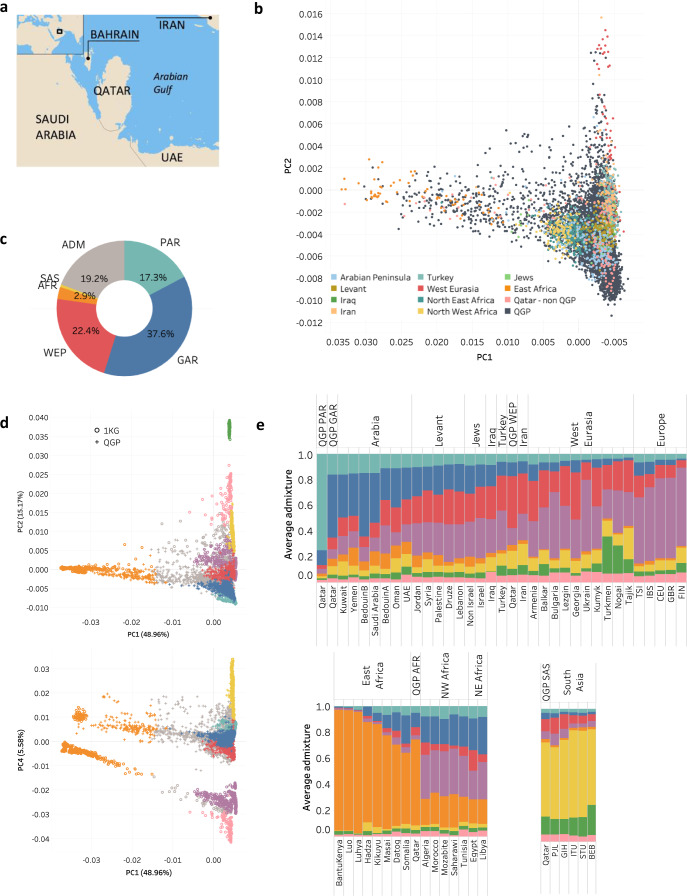
Genetic structure of the QGP population. **a** Map showing the geographical location of Qatar, source of the study population. **b** Principal Component Analysis plot showing overlap of Qatar Genome Program (QGP) subjects with populations from the wider Middle Eastern region found in the Human Origin, Greater Middle East and other public datasets. QGP samples are shown in black and other reference populations in various colors. **c** Genetic sub-groups of the Qatari population based on dominant ancestral fraction (≥0.5) and *k* = 8. The abbreviations refer to Peninsular Arabs (PAR), General Arabs (GAR), Arabs of West Eurasia and Persia (WEP), South Asian Arabs (SAS), African Arabs (AFR), Admixed Arabs (ADM). **d** PCA showing QGP sub-groups in the context of continental populations form Africa, Europe, South Asia, East Asia and America. **e** Average ancestry fractions for QGP and other world populations (*k* = 8). The three sub-panels highlight various reference populations as relevant to the QGP subpopulations. Colors in panels (**c**–**e**) are the same as those used to delineate the distinct ancestral fractions in ADMIXTURE. Abbreviations of the 1KG subpopulations are: BEB Bengali from Bangladesh, CEU Utah Residents (CEPH) with Northern and Western European Ancestry, FIN Finnish in Finland, GBR British in England and Scotland, GIH Gujarati Indian from Houston, Texas, IBS Iberian Population in Spain, ITU Indian Telugu from the UK, PJL Punjabi from Lahore, STU Sri Lankan Tamil from the UK, TSI Toscani in Italy. Source data are provided as a Source Data file.

The Qatari population, with its history and geographical location (Fig. 1a), constitutes a representative subsection of the wider Arab Peninsula^6,12–14^. Here, we analyze the dataset from Phase 1 of the Qatar Genome Program (QGP) which aims to sequence the genomes of the population of Qatar (https://qatargenome.org.qa). We study its genetic structure in the context of regional history of admixture and migration, and build a reference panel to enable better genotype imputation for Arab and related ethnicities. There exist multiple reference panels from large-scale studies which improved imputation accuracy of low-frequency and rare variants^15–19^ (https://www.caapa-project.org; https://www.nhlbiwgs.org; https://imputationserver.readthedocs.io/en/latest/reference-panels/; http://www.hapmap.org; https://imputationserver.readthedocs.io/en/latest/reference-panels) including for specific populations^20–23^. However, they perform poorly for under-represented ancestries such as those from the Middle East^24–26^.

## Results

### Study dataset

We used whole-genome sequencing data on 6218 predominantly healthy individuals recruited from the general population at Qatar Biobank^27^ (https://www.qatarbiobank.org.qa), QGP dataset (Methods). Sequencing was performed using Illumina HiSeq X10 at Sidra Medicine (https://www.sidra.org) achieving a mean coverage of 30x. Joint processing was performed using GATK following its Best Practices Pipeline (https://gatk.broadinstitute.org/hc/en-us/articles/360035894711-About-the-GATK-Best-Practices) and high-quality calls were retained. For the 6218 samples, 74,783,226 Single Nucleotide Variants (SNVs) and 6,560,138 Indels were obtained (Supplementary Table 1), including 28% that were found to be novel (Mbarek et al., In preparation). Autosomal SNVs were further processed resulting in 69,830,209 SNVs (Methods). QC at the sample level resulted in the removal of two samples due to a high level of missingness (Supplementary Fig. 1), thus 6216 were retained. With current standards, this dataset provides a sizeable dataset for a Middle Eastern population. To allow comparison with other populations, we merged our dataset with samples from the 1000 Genomes Project (1KG, *n* = 2504)^28^, The Human Origin project (HO, *n* = 587)^10,29^, the Greater Middle East study (GME, *n* = 731)^6^ as well as previously published whole genomes of Qataris (*n* = 105)^14^ (Methods).

### Population structure of the Qatari population

An assessment of allele sharing between QGP and populations representing various world continental regions, revealed a consistent trend whereby it is highest with Africans (AFR, 22%), followed by South Asians (SAS), Europeans (EUR) and lowest with East Asians (EAS, 9%) (Supplementary Fig. 2). This mirrors the geographical location of the Arab Peninsula relative to the world’s continents. In order to gain more insight into the genetic structure and ancestry of QGP dataset, we performed a number of analyses. First, we conducted Principal Component Analysis (PCA) on the combined samples of QGP, 1KG, HO and GME (Methods). In comparison to continental populations (Supplementary Fig. 3), as described previously^30^, we found PC1 distinguishes 1KG AFR from other populations, PC2 distinguishes 1KG EAS, while lower PCs distinguish 1KG EU, SAS and Americans (AMR). QGP samples form a continuous density that projects on various PCs and is largely separable from 1KG samples. In comparison to Middle Eastern populations (Fig. 1b; Supplementary Fig. 4), QGP dataset is shown to broadly overlap with samples from various regions of Arabia, Levant, Iraq, Iran, Turkey, North and East Africa, which indicates that the QGP population is diverse, showing a good representation of the wider Middle Eastern region.

Next, we ran ADMIXTURE^31^ to estimate the proportions of distinct ancestries on the combined QGP and public datasets. We performed multiple runs of 5-fold cross validation assuming *k* values starting from 2 and found a plateau of low cross validation error from *k* = 3 to *k* = 18 (Supplementary Fig. 5). Two previous studies on Qataris using array data (*n* = 156) and exomes (*n* = 1005) found 3 and 7 ancestral components respectively^12,14^ and a study on 1KG which found 8 components that uniquely described the underlying non-admixed continental populations^16^. In our analysis, consistently we see that starting from *k* = 8 we get distinct ancestral components describing the 1KG populations and this persists with higher *k* values (Supplementary Figs. 6 and 7). For *k* = 8, six main ancestral components were found in QGP: One primarily unique to Qataris and other Middle Eastern subjects (colored in cyan), two are dominant in Qataris and other Middle Eastern subjects and also found in South Europeans, South Asians and Admixed South Americans (blue and red). In addition, QGP has two minor components representing predominantly Africans and South Asians, colored in orange and yellow respectively. One QGP individual is found to have EUR ancestry (purple). Dominant East Asian and Native American ancestries (green and pink respectively) are absent from QGP.

By assigning a major ancestry for each sample based on the dominant ancestral fraction (>0.5), most QGP samples (81%) were found to be grouped to five distinct clusters with major ancestral components (Fig. 1c). We colored them cyan, blue, red, yellow and orange to reflect the dominant ancestral components shown in Supplementary Fig. 7. The other 19% had no dominant ancestral fraction (admixed) and we colored them gray. 77% of QGP samples fall in the blue, cyan and red clusters represented by ancestries that are largely specific to QGP/Middle Eastern populations while 4% fall in the yellow and orange clusters which are represented by ancestries prevalent in South Asian and Africans populations. When overlaid on the PCA, the Cyan, Blue and Red clusters are shown to be distinct from 1KG continental populations (Fig. 1d and Supplementary Fig. 8). Also, QGP samples in the Orange cluster are shown to be closer to East Africans from 1KG relative to Western Africans (Fig. 1d PC1 vs PC4). In addition, QGP Yellow cluster is located closer to 1KG’s Eastern populations of the Indian subcontinent relative to the Western ones (Supplementary Fig. 8, PC2 vs PC3).

By applying higher thresholds for defining dominant ancestry, we obtained smaller non-admixed clusters that are distantly separated by PCA (Supplementary Fig. 9). This impacted QGP and other 1KG populations to various extents, reflecting different levels of homogeneity across populations. Since the 50% threshold objectively defines major ancestry and leads to the largest non-admixed clusters, we use that for subsequent analyses.

Clustering based on *k* = 18, which corresponds to the lowest CV error (0.344) further subdivided the *k* = 8 clusters in a hierarchical manner leading to many clusters with less than 10 subjects (Supplementary Fig. 10). On the other hand, the 1KG subpopulations did not get subdivided after *k* = 8. Since cross-validation errors corresponding to *k* = 8 and *k* = 18 were similarly low (0.347 and 0.344), both *k* values gave robust classifications reflecting different levels of resolution. To obtain a broad insight to population structure and maximize statistical power, we refer below to the main 5 clusters of the QGP populations based on the *k* = 8 analysis unless indicated otherwise.

### Comparison to Middle Eastern populations and nature of QGP ancestries

To determine the nature of the identified QGP ancestry clusters, notably cyan, blue and red, we examined their co-localization on PCA and Admixture sharing relative to publicly available samples from diverse Middle Eastern populations^6,10,13,14,29^. As highlighted in Supplementary Fig. 11, the Blue cluster overlapped largely with samples from Arabia, Levant (including both Arab and Jewish populations) and North Africa. The Red cluster predominantly overlapped with Persians, Turkish and other West Eurasians. Interestingly the Cyan cluster did not overlap with public samples except previously published Qataris^14^ (Supplementary Fig. 11). Consistently, patterns of admixture fractions reflect these relationships and show a number of gradients on the axis from Arabia to Europe (Fig. 1e): (1) Decrease of Cyan and Blue signatures (2) Increase of Purple (dominant ancestry in Europeans) (3) Increase of Red signature towards West Eurasia followed by a decrease towards Europe. We note that for Levant populations, Jewish and Arab populations have similar Admixture patterns reflecting their common ancestral history. The QGP Orange and Yellow clusters have similar signatures to other Eastern African and South Asian populations, respectively. For privacy reasons, it was not possible to get the tribal affiliations of QGP samples, however based on aggregate information we could trace the Cyan cluster to tribes originating from South of Arabia, Blue to the Levant/North Africa and Red to Persia. Therefore, based on these analyses, we name the QGP clusters as: Blue: General Arabs (GAR), Cyan: Peninsular Arabs (PAR), Red: Western Eurasian and Persian Arabs (WEP), Yellow: South Asian Arabs (SAS), Orange: African Arabs (AFR) and Gray: Admixed Arabs (ADM). This further refines the previously described breakdown of the Qatari population into three groups of Bedouins (Q1), Persian/South Asians (Q2) and Africans (Q3)^9,12^. We note that most public samples from Arabia used in our comparison including Bedouins, cluster closer to GAR than to PAR, which suggests their origin in Levant/North Arabia.

### Intercontinental gene flow

**Fig. 2:**
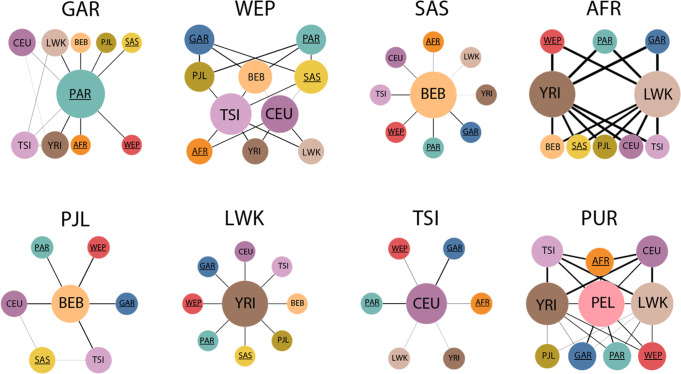
Contribution of Arab populations to various modern continental populations and vice versa. Large headers show populations from QGP (top) and 1KG (bottom) which are a significant target for admixture by other populations from the combined datasets. For each target population, a network is depicted for the underlying pairs of donor populations with a significant F3 statistic (Z < -3). Edge thickness is proportional to absolute Z score and circle size is proportional to the number of connecting edges. Edge length is arbitrary. QGP subpopulations are labeled with an underscore. Abbreviations of 1KG populations are explained in Supplementary Table 2. Source data are provided as a Source Data file.

Genetic distance between QGP subpopulations and 1KG measured using Wright’s Fixation Index (F_ST_) revealed that PAR has the highest degree of differentiation relative to all 1KG populations, especially African (Mean *F*_*ST*_ = 0.148). GAR and WEP show relatively less differentiation versus non-Africans. The QGP African and South Asians are shown to be close to their 1KG counterparts (Mean *F*_*ST*_ = 0.036 and 0.042 respectively) (Supplementary Fig. 12), in agreement with earlier studies^9^. Consistent results were also obtained by calculating genetic drift using TreeMix^32^ (Supplementary Fig. 13). To examine the gene flow involving QGP and 1KG populations, we ran F3 statistics which assesses whether a given target population C was the result of admixture between two source populations A and B^33,34^ (Methods). We tested all possible (A, B, C) combinations for the QGP subpopulations and eight representative populations from Africa (YRI, LWK), Europe (CEU, TSI), South Asia (PJL, BEB), and America (PUR, PEL) (See Supplementary Table 2 for explanation of 1KG populations abbreviations). Target populations with significant contributions (F3 < 0 and Z < -3) are shown in Fig. 2. We found that PAR was not a significant target of admixture for any population while it is a source for many, unlike other QGP populations which act both as source and recipient of admixture. PAR was the dominant source for GAR in combination with WEP, South Asian and African populations (Z -18.3 – -27.5). WEP has admixture coming from PAR/GAR with South Asian populations on one side and European with African in another (Z -21.6 – -42.4). QGP SAS had BEB as the main donor of admixture particularly in combination with non-African QGP and Europeans (Z -14.7 – -23.1). QGP AFR show the highest Z scores in the analysis, receiving admixture from both Eastern and Western African populations in combination with various QGP, European and South Asian populations (Z -91.5 – -119.4). In addition, we also found that QGP populations were significant donors to other continental populations from East Africa (LWK), Europe (TSI), South Asia (PJL) as well as admixed Americans (PUR), which extends previous findings^35–37^.

### Effective population size and divergence history

**Fig. 3:**
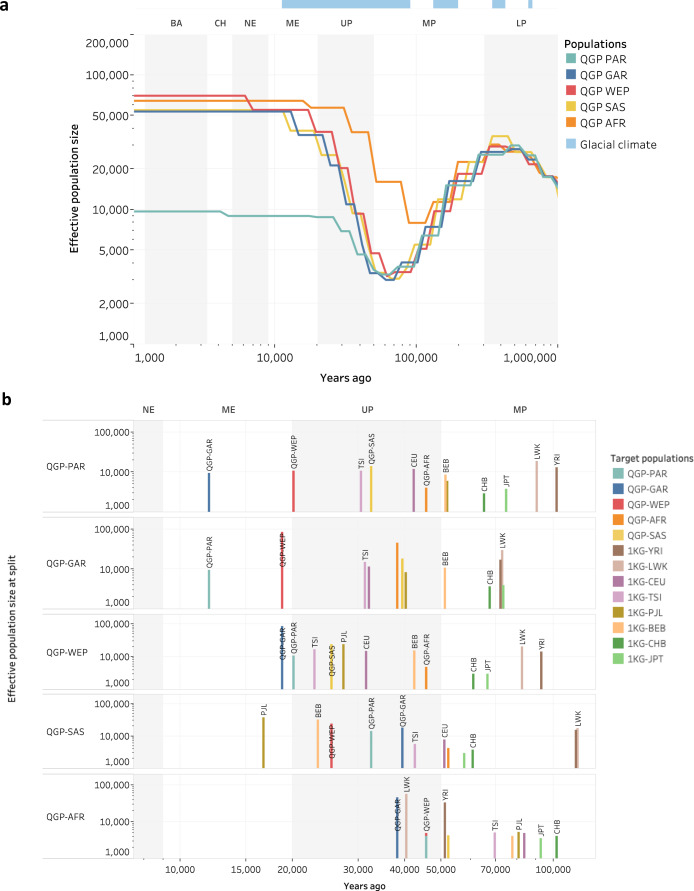
Demographic history of the QGP subpopulations. **a** Effective population size over time, inferred using SMC++. **b** Estimated split times for each Qatari subpopulation relative to other Qatari and representative populations from Africa, Europe and South Asia and East Asia, indicating the ancestral population size at the time of split. Archeological periods are highlighted with alternating gray and white backgrounds and labeled as LP (Lower Paleolithic), MP (Middle Paleolithic), UP (Upper Paleolithic), ME (Mesolithic), Neolithic (NE), Chalcolithic (CL), Bronze Age (BA). Glacial periods are indicated horizontally on the top. Abbreviations of 1KG populations are explained in Supplementary Table 2. Source data are provided as a Source Data file.

No studies to date applied coalescence methods to estimate the time of divergence of Arab populations and few estimated their effective sizes going back hundreds of thousands of years ago using a small number of genomes^9^. Here we leverage the large size of the dataset and use SMC++, a scalable approach that combines information from both sequential Markovian coalescent and site-frequency-spectra based frameworks^38^ to infer effective size history and divergence time of the QGP population. We run that on hundreds of representative genomes per subpopulation for QGP and eight world populations from Africa, Europe, South Asia and East Asia (Methods). As depicted in Fig. 3a, QGP non-African subpopulations experienced the characteristic bottleneck around 70 kya similar to other non-African populations^38,39^ (Supplementary Fig. 14a). All populations recovered during the Upper Palaeolithic period, however while GAR, WEP and SAS continued to expand and then stabilized in the Neolithic period (which is marked by the beginning of farming in the Near East), PAR levelled off earlier around 21 kya. This time period corresponds to the beginning of aridification in Arabia^40^, suggesting by that time GAR, WEP and SAS had spread to more fertile regions in North Arabia/Levant while PAR remained largely in the drier Middle/South of Arabia where they continued their hunter-gathering lifestyle.

Split time analysis shows that PAR, GAR and WEP have a common founder population, with PAR and WEP splitting 20 kya while PAR and GAR splitting 12 kya (Fig. 3b). This was concurrent with the start of aridification in Arabia and that of farming in the greater Levant, respectively. PAR and GAR had similar split times with various European and South Asian populations which highlights their closeness. Notably, the split between PAR and Africans (YRI, LWK) happened at similar time as between Europeans/South Asians and Africans (around 90 kya). This supports out of Africa migration via Arabia and is consistent with *Homo sapiens* fossil being found in Arabia dating back 85 kya^41^. Later on, PAR split from European 42 kya and then South Asian population around 32 kya. Interestingly, the QGP sub-African population split early from other European and South Asian populations, similar to LWK and YRI. However, it split with YRI and LWK 40–50 kya which is relatively close to its latest split with a non-African population (GAR, 38 kya) and is much earlier than the split between LWK and YRI (16 kya). While the QGP African group may reflect admixture due to recent slave trade, its African ancestry seem to have diverged relatively early from both African and Arab populations.

### Shared ancestry with ancient human populations

**Fig. 4:**
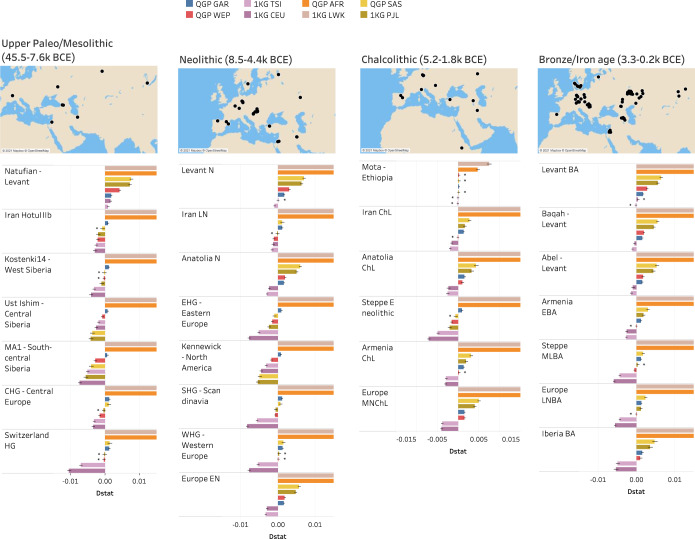
Shared ancestry with ancient human populations from various archeological periods. Bar plots showing D-statistic for the contribution of various ancient human genomes to PAR ancestry relative to other QGP and world populations, inferred with Patterson’s D-statistic (Dstat). Results are grouped by archeological periods. The maps show the geographical locations of the corresponding ancient genomes. The dates refer to estimated time range of the ancient genomes (Dates for individual genomes are found in Supplementary Material). D-statistics values with low absolute Z score (<3) are highlighted with *. Black lines at the end of bars indicate 95% confidence intervals. Negative D-statistic value imply higher introgression with PAR relative to other tested populations while positive values imply the opposite. For clarity, bars for QGP AFR and 1KG LWK are shown up to 0.01 (They extend to maxima of 0.03 and 0.05 respectively). Numbers of samples used for QGP are AFR *n* = 179, GAR *n* = 2338, SAS *n* = 40, WEP *n* = 1390 and for 1KG are CEU *n* = 99, LWK *n* = 99, PJL *n* = 96, TSI *n* = 107. Abbreviations of 1KG populations are explained in Supplementary Table 2. Source data are provided as a Source Data file.

To put the QGP ancestries in the context of ancient human populations, we performed D-statistics analysis^34^ using the QGP dataset against 352 published ancient human genomes previously discovered in Africa, Levant, Iran, Anatolia and Europe dating back to various archeological periods ranging from Upper Paleolithic to Iron age^10,42,43^ (Supplementary Fig. 15). As described in Methods, we compared the contribution of various ancient human genomes to PAR as a baseline, relative to other QGP and world populations (Fig. 4). For all ancient populations, regardless of time, we consistently see that African populations have significantly less ancient DNA than PAR. In the Levant region, PAR was shown to contain more ancient DNA ancestry than any QGP, African or Asian population across various archeological periods (Z-score <3). In comparison to European populations, PAR had more Natufian ancestry during the Mesolithic period, then over time, they show less ancient Levant ancestry (Fig. 4 and Supplementary Fig. 16). In other regions outside the Levant, PAR has less ancient DNA in comparison to other populations in all periods, except for the Chalcolithic Mota in Ethiopia, where PAR had similar amounts to non-African populations, which supports back migration to Eastern Africa^44^. Furthermore, we saw a trend of increasing European, West Eurasian and South Asian ancestry relative to PAR in ancient genomes along the northern route of migration from Levant towards the Steppes, Central Asia and Northern Europe. On the southern route of migration, between Arabia and Europe, we see that PAR ancestry is only dominated by European and is still higher than West Eurasian and South Asian. Consistently, the Asian ancestry shows the largest sharing with Kennewick ancient genome from North America. GAR has systematically the least difference to PAR in ancient admixture, highlighting the relatively more recent split of these populations. Overall, these results are consistent with the expansion of ancient farmers from the Near East to Europe^10^ and points to the PAR amongst other Arab populations to represent the indigenous Arabian population that descended from the first Eurasian populations established by out of Africa migrations^9^. Importantly, these results show that ancient indigenous Arabs lived in the fertile crescent of the Levant in addition to other parts of Arabia where numerous paleolithic environmental sites have been found^40^.

### Inbreeding and runs of homozygosity

**Fig. 5:**
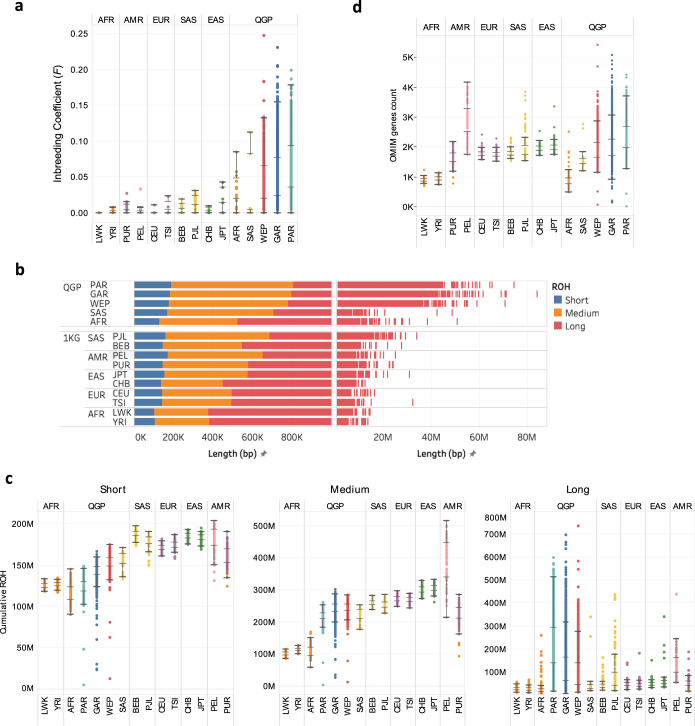
Runs of homozygosity and relatedness in QGP in the context of world populations. **a** Distribution of inbreeding coefficient (*F*) in QGP and reference world populations. **b** Distribution of ROH segments ordered and colored by size class, highlighting a shift in ROH class boundaries towards longer ROH for medium and long classes for the QGP subpopulations in comparison to the 1KG populations. **c** Cumulative size of short, medium and long ROH segments across individual samples based on Gaussian mixture model clustering highlighting considerably more samples enriched for long ROH in QGP PAR, GAR and WEP subpopulations. Populations are sorted by ascending median of cumulative medium ROH. **d** Count of OMIM genes that completely overlap with ROH per population. Populations are ordered as in **a**. Numbers of samples used for QGP are AFR *n* = 179, GAR *n* = 2338, PAR *n* = 1073, SAS *n* = 40, WEP *n* = 1390 and for 1KG are BEB *n* = 86, CEU *n* = 99, CHB *n* = 103, JPT *n* = 104, LWK *n* = 99, PEL *n* = 85, PJL *n* = 96, PUR *n* = 104, TSI *n* = 107, YRI *n* = 108. Boxes indicate median and middle two quartiles of the data. Whiskers indicate data 1.5 times the interquartile range. Abbreviations of 1KG populations are explained in Supplementary Table 2. Source data are provided as a Source Data file.

Middle Eastern populations have relatively high levels of consanguinity (20–50% versus <0.2% in western Europe and Americas)^4,45^, resulting in the accumulation of long Runs of Homozygosity (ROH). These ROH regions are of medical interest because they can be associated with autozygosity of deleterious founder mutations^8,46,47^. In terms of population structure, short, medium and long ROH respectively reflect ancient haplotypes predating continental migration, background relatedness and recent parental relatedness^48,49^. The QGP cohort was not targeted for specific family relationships, therefore it is likely to represent the sampled population. Consistent with previous observations, QGP had significantly higher inbreeding coefficients (*F*) relative to other world populations (Fig. 5a). This is most prominent for PAR (median *F* = 0.060 versus 0.008–0.014 for 1KG). Next, we leveraged the dataset to perform ROH analysis at large scale using whole-genome sequencing data, which is known to be more accurate than based on exome data^50^. We called ROH for each individual and run a Gaussian Mixture model per population to classify it to short, median and long ROH classes^48^, which is more accurate than adopting class boundaries form Caucasian populations as done in Scott et al.^6^. (Methods, Supplementary Fig. 17). This identified population-specific boundaries for the three classes, showing an upward shift in QGP relative to reference continental populations (Fig. 5b).

By examining cumulative ROH length, QGP subpopulations are shown to have the least short and medium ROH, reflecting their closest link to the early populations that migrated out of Africa (Fig. 5c). On the other hand, PAR, GAR and WEP have high proportions of long ROH reflecting their recent interbreeding. While short and medium ROH correlated across various populations, there was a negative correlation between long and short ROH in particular in PAR, GAR and WEP (*R* = 0.55–0.78; *p* < 0.0001) (Supplementary Fig. 18a). Cumulative length and count of ROH correlated well for both short and medium ROH (*R* > 0.9; *p* < 0.01) unlike for long ROH. Despite having higher cumulative long ROH, PAR, WEP and GAR have lower long ROH counts than non-admixed Americans, which highlights the background relatedness caused by historical isolation of the latter population. In comparison with previous studies on Middle Eastern populations such as Scott et al.^6^, we saw longer ROH fragments (exceeding 80 MB), which is likely due to the larger sample size and the use of WGS versus exome/array data^47,50^. In line with the trend of cumulative total ROH, the median count of OMIM genes overlapping with ROH is generally lowest among African populations and highest among non-admixed American populations. QGP showed the largest spread whereby individuals are found with more than 4000 ROH-overlapping disease genes (Fig. 5d), which extends earlier findings^5^.

### Mitochondria DNA and Chromosome Y haplogroups

**Fig. 6:**
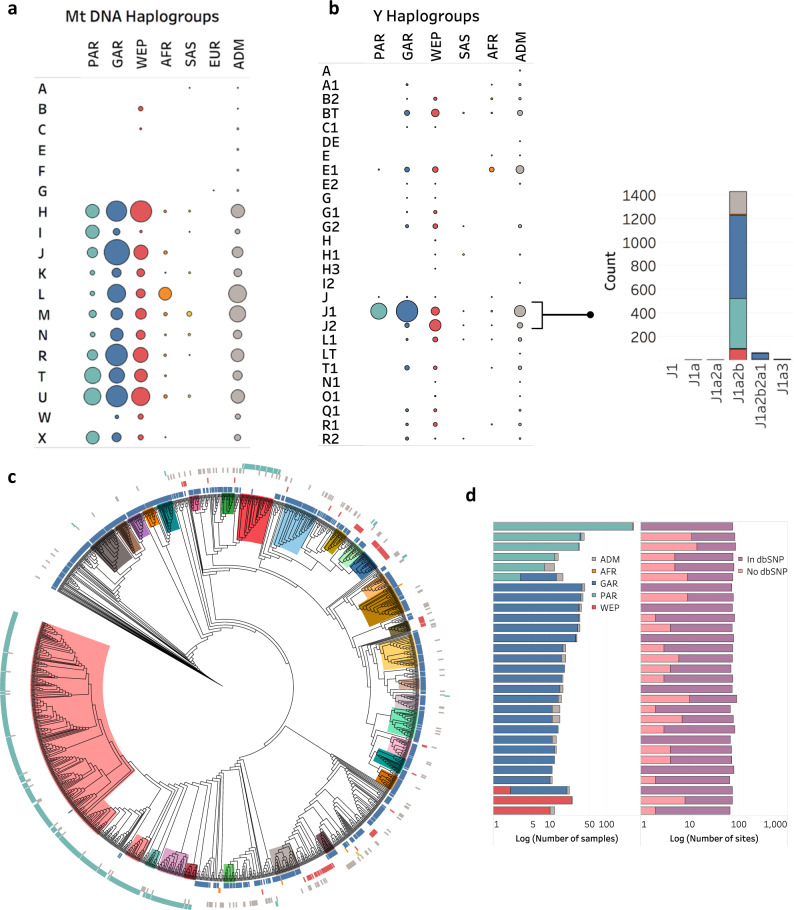
Chr Y and mtDNA haplogroups of QGP samples. **a** mtDNA haplogroup and **b** Chr Y assignments in the various QGP subpopulations. Number of samples is indicated by circle size. Inset shows breakdown for J1 clade. **c** Maximum likelihood tree for samples assign ed to J1a2b Y haplogroup (bootstrap > 90%). Clusters are defined at genetic distance cutoff 5 × 10^−4^. Outer circles indicate autosomal ancestries (QGP subpopulations) and are colored accordingly. Clusters inside the tree are colored independently based on the identified 29 unique sub-haplogroups. **d** Partitioning of the 29 sub-haplogroups amongst autosomal ancestries and numbers of underlying SNVs indicating presence in dbSNP build 151. Source data are provided as a Source Data file.

In recent years, studies on chromosome Y (Chr Y) and mitochondrial DNA (mtDNA) gave useful insights into world population migration, including tracing the origin and expansion from the Middle East^51,52^. Consistent with known mtDNA maps^53^, we saw that QGP AFR and SAS have predominantly L and M haplogroups respectively, while the other QGP subpopulations have more diverse haplogroups with PAR being the least heterogeneous (Fig. 6a and Supplementary Fig. 19). U is largely specific to PAR, GAR and WEP. U9, X2 and I (mostly I7) are found almost exclusively in PAR. U3 has been seen before along the Arabian sea coast while X is found in Anatolia region^54,55^.

In contrast to the observed mtDNA diversity, and in line with patrilocal practices in the region, 56.7% of male subjects have J1 Chr Y haplogroup clade (*n* = 1524) (Fig. 6b). J1 is known to be prevalent in Levant and Arabian Peninsula, particularly in Yemen (72%)^51^. This haplogroup is almost universal among PAR (99.1%), abundant in GAR (77.0%) while it is modestly present in WEP (16.7%), SAS (12.5%) and AFR (7.9%). Most subjects in this J1 clade were assigned the J1a2b haplogroup (*n* = 1426, 93.6%) (Fig. 6b inset and Supplementary Fig. 20). Thus, the QGP dataset comprises the largest set of individuals reported to date with J1a2b^51,56–58^. In terms of other haplogroups, consistent with previous work, J2 is largely confined to WEP (80%) while E1 and H1 are most dominant in AFR (58.3%) and SAS (31.3%) respectively. Homogeneity of PAR individuals supports the uniqueness of this subpopulation suggested by the earlier autosomal ancestry analysis (Fig. 1). Overall, these findings highlight the migratory history of Arab Peninsula and Levant regions, the presence of strong barriers to inter-marriage outside tribal groups and the dominance on gene flow of female movement from other geographical regions to Arabia^9,12,51^.

### Novel sub-haplogroups for J1a2b and lineage dating

We further characterized the set of 1426 samples assigned to haplogroup J1a2b by generating a bootstrapped Maximum-likelihood phylogenetic tree (Fig. 6c). We clustered samples in the tree by applying a range of genetic distance cut-offs from 1 × 10^−5^ to 4 × 10^−3^ and searched for a cut-off that resulted in the maximum number of large clusters (sample size >10) (Supplementary Fig. 21). The largest number of such clusters (*n* = 31) was obtained using a 5 × 10^−4^cut-off. In total, 331 informative SNPs were found to distinguish these clusters based on F_ST_ = 1 (Supplementary Data 1). These SNPs, 103 of which were not previously reported in dbSNP, define 29 unique J1a2b sub-haplogroups which partition well among the autosomal ancestry clusters (Fig. 6c, d). The estimated time of divergence of these sub-haplogroups ranges from 13.2 kya to 9.4 kya, which is consistent with the estimated time of divergence between PAR and GAR based on the autosomal split time analysis with SMC++, given that most of these haplogroups are found in these two populations. These sub-haplogroups can be divided into shorter segments by applying lower genetic cut-offs for defining the clusters, which is expected as clusters are further subdivided in this process (Supplementary Fig. 22).

### Imputation panel

**Fig. 7:**
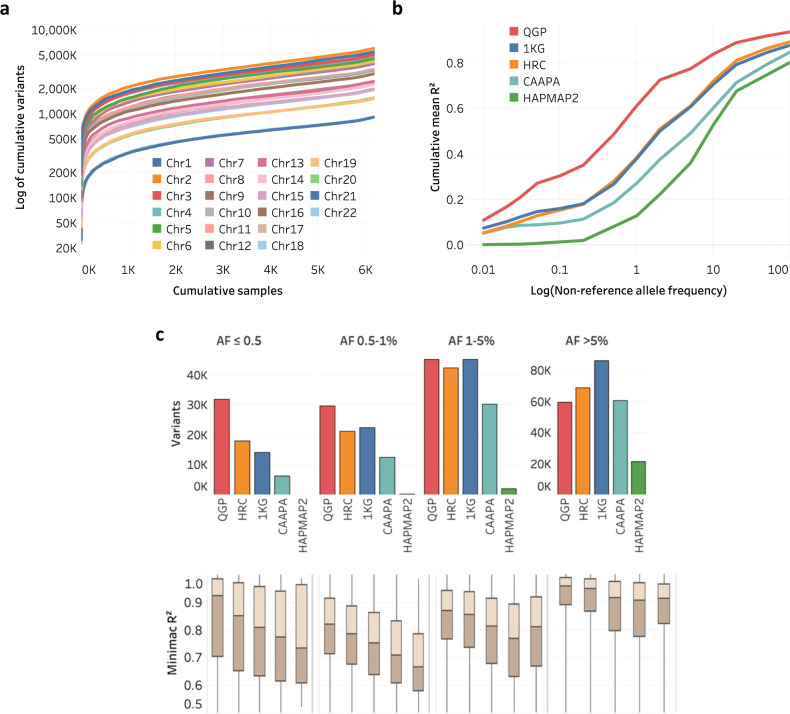
Imputation using QGP versus other publicly available reference panels. **a** Cumulative variants (log of SNV count) discovered in the QGP phase 1 dataset as a function of cohort size. **b** Imputation performance of QGP panel versus panels of HRC, 1KG, CAAPA and HAPMAP2(https://imputationserver.readthedocs.io/en/latest/reference-panels). Shown is imputation accuracy measured by cumulative mean *R*^2^ when imputing SNP genotypes into 105 independent Qatari samples as a function of logarithm of non-reference allele frequency of imputed SNPs. The results are based on genotypes on Affymetrix 6 array as a pseudo-array data. **c** Number of imputed variants using various panels per category of predicted minor allele frequency (top) and respective distribution of high quality Minimac *R*^2^ scores (>0.5) (bottom). Number of samples tested is *n* = 105. Boxes indicate median and middle two quartiles of the data. Whiskers indicate the range of the data. Source data are provided as a Source Data file.

Current publicly available imputation panels lack adequate representation from Arab and Middle Eastern populations^26^. We therefore built an imputation panel based on the QGP dataset to improve imputation for these populations. We first assessed the extent to which the current cohort provides an adequate set of variants for the relatively small Qatari population (Fig. 7a). As a function of cumulative samples in the QGP dataset, the number of new variants discovered was found to increase rapidly for the first 300 samples and continues approximatively logarithmically with sample size (*p* value < 10^−4^) by an average of 2–3% per 100 samples. Therefore, the current cohort would still benefit from sequencing additional samples, yet it is adequately sized to capture variants common in this population for the purpose of imputation. To build this panel, we used the high-quality set of 68,107,887 autosomal SNVs for 6216 individuals obtained by filtering out variants with missingness >1% and HWE *p* value < 10^−5^. We did not include short indels at this stage as they are generally less reliable and require validation. Using these SNVs, phased haplotypes were produced and converted to Minimac 3 format^59^. This QGP panel has some key features. Unlike many of the large publicly available panels, all samples were collected, sequenced and processed in the same site and show consistently high coverage. Also, it has relatively high diversity. While having ~80% less samples than the Haplotype Reference Consortium (HRC) panel, it contains almost double the number of variants, and that is only marginally fewer than the worldwide 1KP panel (Supplementary Table 3).

Performance was assessed for imputing variants from an unrelated set of 105 diverse Qatari whole genomes that were published previously^14^, against the reference panels of QGP, 1KG^16^, HRC^19^, CAAPA (https://imputationserver.readthedocs.io/en/latest/reference-panels) and HAPMAP2 (http://www.hapmap.org). From the known whole-genome genetic variation of these individuals, we created three pseudo arrays with different marker densities based on CoreExome (https://www.well.ox.ac.uk/~wrayner/strand/humancoreexome-12v1-1_a-b37-strand.zip), Affymetrix 6.0 (https://www.well.ox.ac.uk/~wrayner/strand/GenomeWide_6-b37.58-v2.zip) and Illumina 5 m (https://www.well.ox.ac.uk/~wrayner/strand/HumanOmni5-4v1-1_A-b37-strand.zip). Genotypes imputed using these pseudo arrays based on the various panels were compared to the known genotypes and level of concordance (*R*^2^) was calculated for multiple bins of alternative allele frequency (AF) (Fig. 7b and Supplementary Fig. 23). The QGP panel is shown to improve imputation performance across all ranges of AF notably for ultra-rare, rare and less common variants (<1%) where mean *R*^2^ is higher by as much as 61.1% relative to second best panel. Also, the number of high-quality imputed variants (Minimac imputation score > 0.5) is generally higher by using the QGP panel (by 57.1% for AF < 1% versus HRC and by 30.9% for AF < 5% versus 1KG) and so are the respective Minimac imputation scores (Fig. 7c). HRC and 1KG have comparable performances, but the latter is slightly better for lower AF and slightly worse for higher AF, reflecting the fact that 1KG variants are largely included in HRC except for very rare variants^19^.

## Discussion

This study provides an in-depth analysis of the genomic structure of the QGP phase 1 cohort, representing a comprehensive set of genomes from a Middle Eastern population. Despite the relatively small size of Qatar, it revealed unique and shared ancestries reflecting the wider Middle Eastern region and its centrality to recent and distant history.

The large dataset allowed the refinement of previously described genetic ancestries in the Qatari population (Q1, Q2 and Q3)^12^, identifying five main distinct ancestries that are generalizable to Arab and other Middle Eastern populations (PAR, GAR, WEP, SAS, AFR). PAR, GAR and WEP were found to be descendants of the main Arab genealogical branches of Qahtanite and Adnanite, which refer to indigenous Arab and Arabized populations respectively. The terms ‘Arab Bedouin’ and ‘indigenous Arab’ were used interchangeably in previous studies, however we find that in many cases this is inaccurate. Modern gene flow was identified from these ancestries to various European, South Asian and South American populations, likely reflecting post-Islamic expansion.

We performed an estimation of time divergence of Arab populations using hundreds of genomes. This pointed to an ancestral Arab population that dominated the Levant region, and split into modern lineages around 12–20 kya. Analysis involving ancient human DNA dating back to various archeological periods indicated that Peninsular Arabs are ancestral to modern Middle Eastern ancestries, being the closest to the basal founders that populated the ancient Near East. The sequencing of ancient genomes from the Arabian Peninsula will shed more light on the contribution of native Arabs to early out-of-Africa migrations.

In addition to enhancing the resolution on population structure, we performed ROH analysis using whole-genome data for a Middle Eastern population and at large scale, identifying some of the highest levels of human ROH ever reported to date. This highlights the tribal, endogamous nature of Arab society and culture, as well as the utility of next-generation sequencing to uncover recessive founder pathogenic alleles in high consanguinity setting^8^. Population-specific boundaries defining various ROH classes were calculated, showing an upper shift in Arab in comparison to world populations. Arabs also exhibited the least short ROH after African populations, reflecting their proximity to out-of-Africa migrations.

Furthermore, we report a substantial number of sequenced individuals having the J1a2b haplogroup which we further characterize into 29 novel sub-haplogroups. These sub-haplogroups were found to partition well among the autosomal ancestries, reinforcing the tribal, patrilocal nature of the regional populations.

Finally, a dedicated QGP imputation panel was generated to leverage this dataset, which shall complement the currently available panels by providing more accurate imputation of Arab and Middle Eastern genomes. This will enable association studies with greater scale and statistical power to detect causal variants underlying biological traits and diseases. Notably, there is an ongoing effort to build a population genotypic array (Q-chip) that would leverage rare and novel missense/LoF variants in disease genes from this panel (Rodriguez-Flores et al. In press). The current dataset has recently helped identify novel loci associated with a range of clinically relevant traits^60,61^. The value of this resource will expand in the upcoming phases of QGP as tens of thousands of additional subjects will be sequenced over the next few years, enabling vital future genomic and medical research in the Middle East and globally.

## Methods

### Cohort description

The current study used data related to peripheral blood samples for 6218 adult subjects (age ≥ 18) collected as part of Qatar Genome Project (QGP) phase 1. These individuals were Qatari nationals randomly recruited from the general population at Qatar Biobank. They all signed an informed consent form prior to their participation, and the study was approved by Hamad Medical Corporation Ethics Committee and QBB institutional review board. Their samples were transferred to Sidra Medicine where their genomic DNA was extracted and used to perform whole-genome sequencing.

### Genome sequencing and variant calling

DNA extraction was performed on Qiasymphony automatic system (QIAGEN). Standard library preparation was done for all samples using a PCR-free sample preparation. Libraries were sequenced on Illumina HiSeq X Ten (Illumina, San Diego, CA, USA) according to the manufacturer’s recommendations. 150 bp paired-end reads were used to the mean coverage of 30x with a median insert size of 400 bp ± 25%. Data generation used HiSeq X Illumina Software v3. Sequencing reads were aligned to GRCh37/hg19 human reference genome using BWA-MEM v0.7.7^62^. BAM files were compressed from SAM then sorted and indexed respectively using view, sort and index functions in SAMtools v1.2^63^. PCR duplicates were then marked using Picard v1.117 MarkDuplicates.jar (http://broadinstitute.github.io/picard). Genome Analysis Toolkit (GATK) v3.4^64^ was used to do further BAM improvements, including realignment around known indels and base quality score recalibration. Single-sample genotypes were called by GATK HaplotypeCaller algorithm (-ERC GVCF). All gVCF-files were combined (-T CombineGVCFs) and jointly called (-T GenotypeGVCFs).

### Quality control

The original set of 6218 samples yielded sufficient input DNA (>1.2 ug) and had successful library preparation and low contamination rate (<10%).

GATK Variant Quality Score Recalibration was performed on the variants and only Single Nucleotide variants (SNVs) annotated as PASS were considered in further analysis.

Regarding sample QC, per sample missingness was calculated using vcftools v1.9^65^. All samples were found to have total missingness levels <0.05 except for one sample that had missingness of 0.81. Per chromosome missingness identified an additional sample which had missingness level of 0.52 for chromosome 18. These two samples were excluded from further analysis. rtg-tools v3.8 (https://www.realtimegenomics.com/products/rtg-tools) was used to calculate the number of variants, non-reference variants (ALT), heterozygous variants (HET), HET/(HET + HOM) ratio, transition/transversion (TITV) ratio per sample in order to identify any outlier samples (below or above 3 SD from the population mean). Also, genotype and phenotype sex concordance were checked for each sample. The final set that was retained contained 6216 samples.

With regard to variant filtering, variants with missingness rate of >1% were excluded. In addition, we filtered out variants with minor allele count ≤2, Hardy–Weinberg equilibrium test *P* value < 10^−5^ and variants in low-complexity regions. At the end we obtained a clean set of 69,830,209 variants for downstream analysis.

### Public datasets of modern populations

Datasets used for comparison are from 1KG project^28^, Human Origin dataset^10,29^, GME^6^, Kuwaiti^13^ and Qataris from non-QGP^14^. HO contains 587 individuals genotyped with Affymetrix array from the Middle Easter region including Arabian Peninsula (*n* = 58; Bedouin, Saudi Arabia, Yemen), Levant (*n* = 122; Druze, Jordan, Lebanon, Palestine and Syria), Turkey (*n* = 56), Iran (*n* = 46), North East Africa (*n* = 23; Egypt and Libya), North West Africa (*n* = 52; Tunisia, Algeria and Morocco), various Jewish communities (*n* = 67) in addition to West Eurasia (*n* = 104) and East Africa (*n* = 59). GME has 731 exomes from Arabian Peninsula (*n* = 161; Kuwait, Oman, Qatar, Saudi Arabia, UAE and Yemen), Levant (*n* = 23; Jordan, Lebanon, Palestine and Syria), Turkey (*n* = 138), Iran (*n* = 55), Iraq (*n* = 5), North East Africa (*n* = 251; Egypt and Libya), North West Africa (*n* = 88; Tunisia, Algeria and Morocco) and Jews (*n* = 10).

### Principal component analysis

Principal Component Analysis (PCA) was performed using PLINK v2.0^66^ on SNVs shared between QGP and 1KG, those shared between QGP, 1KG and other reference samples from Human Origin dataset^42^, Greater Middle East dataset and previously published Qatari genomes^14^, and those shared between the later and Kuwaiti^13^. In the three cases, the shared SNVs were filtered based Linkage Disequilibrium (LD) cut-off 0.2, resulting in 14,396,599, 151,981 and 4238 variants respectively. Next, these were used as input for running PLINK to calculate the PCs. Given the diversity of the study samples, we did not exclude low frequency variants since they were shown to help with stratification^67^.

### ADMIXTURE analysis

To run ADMIXTURE v1.3.0^68^, we intersected SNVs common to the datasets using bcftools isec (26,393,812 variants) and filtered them based on Minor Allele Frequency (MAF) 0.05 and LD 0.2 using PLINK in order to achieve efficient computation time. This resulted in 351,181 variants which we used as input to run ADMIXTURE with five-fold cross validation by assuming various *k* values.

### TreeMix analysis

TreeMix analysis was performed on the combined QGP and 1KG datasets using TreeMix v1.3^32^. Starting from the VCF file containing the intersection set between QGP and 1KG pruned at LD 0.2 (14,396,599 variants), the input file for TreeMix was generated using glactools v1.0^69^. To meet the tool’s requirement, multiallelic sites were split and missing sites were replaced with reference. TreeMix was run using block size of 500 and bootstrap option, allowing for 0–5 migrations each in a separate analysis. The resulting maximum likelihood trees were plotted using iTOL (https://itol.embl.de).

### F3 statistics analysis

F3 statistics test was used to assess genetic contribution of QGP populations to various modern world populations and vice versa^33,34^. For that we run qp3pop as part of ADMIXTOOLS v7.0.1 using default parameters^34^. F3 statistics test takes two source populations (*A, B*) and one target population *C*. The method estimates allele frequency correlations between *A* and *B* relative to *C* and by calculating the product of allele frequency differences between population *C* to *A* and *B*. A negative F3 statistics value suggests that *C* is indeed admixed with population *A* and *B*. The method also outputs a Z score as the deviation of the F3 statistic from zero in units of the standard error. Absolute Z > 3 suggests significant rejection of the null hypothesis that F3 statistics is not negative. We ran the F3 statistics test using all possible three-population combinations from the QGP subpopulations (GAR, QGP AFR, WEP, PAR and QGP SAS) and eight representative 1KG populations (YRI, LWK, CEU, TSI, PJL, BEB, PUR, and PEL).

### Effective population size and divergence history

We estimated population size trajectories and divergence time between populations using SMC++ v1.15.4^38^. This was run using multiple genomes per Qatari subpopulations (PAR, *n* = 201; GAR, *n* = 201; WEP *n* = 201, *n* = 201; AFR, *n* = 179; SAS, *n* = 40) and eight 1KG populations representing Africa (LWK, *n* = 99; YRI, *n* = 108), Europe (CEU, *n* = 99; TSI, *n* = 107), South Asia (PJL *n* = 96; BEB, *n* = 86) and East Asia (CHB, *n* = 103; JPT, *n* = 104). As recommended by the software’s authors (https://github.com/popgenmethods/smcpp) we first ran *vcf2smc* subcommand per population to convert SNP data for chromosomes 1 to 22 to SMC++ format, masking centromeres. This was followed by *estimate* subcommand which calculates for each population the effect population size as a function of time. For that, we used per-generation mutation rate of 1.25e−8, piecewise spline, knots 24 and timepoints in generations as *t*_1_ = 33 and *t*_n_ = 34,000. Next, we created a SMC++ pairwise population dataset using the *vcf2smc* subcommand masking the centromeric region as before. Using the per population SMC++ data and JSON file from *estimate*, in addition to the pairwise population SMC++ data, we ran the *split* subcommand with timepoints *t*_*1*_ = 33 and *t*_*n*_ = 34,000 to generate the pairwise population JSON file. The *split* subcommand fits two-population clean split models using marginal estimates produced by *estimate*. The *plot* subcommand was used to generate a CSV file form the obtained output which we plotted using internal scripts.

### Admixture with ancient human populations

Ancient human genomes data from previously published studies (*n* = 352)^10,42,43^ was downloaded in Eigenstrat format from https://reich.hms.harvard.edu/sites/reich.hms.harvard.edu/files/inline-files/NearEastPublic.tar.gz, and combined with QGP data. This was used as input for qpDstat program in ADMIXTOOLS v5.1^34^ to calculate Patterson’s D-statistic which assesses the contribution of ancient human genome ancestries to QGP PAR relative to QGP and 1KG populations representing Europe (TSI, CEU), Africa (LWK) and South Asia (PJL). The D-statistic compares counts of the derived ancient genomes alleles versus distant outgroup alleles (Chimpanzee) in QGP PAR and a comparison population from QGP and 1KG. D-statistic calculation requires four populations: a comparison population (*w*), a basal population (*x* = QGP PAR), an ancient population (*Y*) and a distant outgroup (*Z* = Chimpanzee). Excess of *Y* alleles in *W* compared to PAR results in a positive D-statistic, meaning higher ancient ancestry in *W* compared to PAR, and vice versa. The higher the D-statics the higher the significance. Also, Z-scores are calculated whereby D-statistics with absolute Z > 3 is generally considered significant.

### Runs of homozygosity

Runs of homozygosity (ROH) were calculated using BCFTools v1.9 (with RoH extension) which uses a hidden Markov model optimized for whole-genome sequencing data^50^. We applied Phred score >50 in order to retain high confidence ROH segments for downstream analysis. Per population, we classified ROH segments into short, medium and long size classes by applying a Gaussian mixed model on density distribution of ROH length using mclust R package^70^, following the recommended approach^48,49^. This identified population-specific boundaries for the three ROH classes, consistent with other studies. Short, medium and long ROH reflect ancient haplotypes that predate continental migrations, background relatedness within populations and recent parental relatedness, respectively.

### Assignment of mitochondrial DNA and chromosome Y haplogroups

A version of GATK supporting haploid chromosome calling (based on v3.4)^64^ was used to call SNPs and Indels from mitochondrial DNA (mtDNA) and chromosome Y (Chr Y) for all QGP samples (*n* = 6216) and QGP males only (*n* = 2687), respectively. SNPs only were retained for downstream analysis. Multiallelic sites were split and variants with missingness levels > 1% were excluded. mtDNA haplogroup assignments were made using HaploGrep v2.1.20^71^ which relies on PhyloTree catalog build 17^72^. Chr Y assignments were made using yhaplo v1.0.18 (https://github.com/23andMe/yhaplo) which uses reference haplogroups from the International Society of Genetic Genealogy^73^.

### Identification sub-haplogroups for Chr Y J1a2b haplogroup

In order to further characterize the set of 1426 samples that were assigned to J1a2b haplogroup, we run RaxML v8.0 which performs Maximum-likelihood based phylogenetic inference^74^. For that, we focused on a 10-Mb region on Chr Y known to be amenable to analysis based on short read sequencing, excluding singletons and SNPs with missingness levels >1%^75,76^. vcf2phylip v2.3 (https://github.com/edgardomortiz/vcf2phylip) was used to convert VCF to FASTA format which was used as input to run RaxML using 100 rapid bootstrap searches and 10 maximum likelihood searches.

Clusters within the resulting phylogenetic tree were defined using ClusterPicker v1.2.3^77^ by applying multiple genetic distance cut-offs ranging from 1 × 10^−5^ to 4 × 10^−3^. The lower and upper boundaries of this range produced the maximum and minimum number of non-singleton clusters, respectively. Branches supported by Bootstrap values <0.9 were collapsed. Trees were plotted using iTOL 9 (https://itol.embl.de).

For each genetic distance cut-off, informative SNPs fixated to individual clusters were identified by calculating *F*_*ST*_ score using a version of vcftools that supports haploid genotypes (v.0.14)^65^ and selecting SNPs with *F*_*ST*_ = 1.

A haplogroup was defined for each non-singleton cluster as the list of SNPs with *F*_*ST*_ = 1 and AF = 1 in the respective cluster.

Lineage dating for each haplogroup was calculated using the method of Poznik et al.^78^, which takes into account the number of SNPs for a given haplogroup, mutation rate and average number of years per human generation. Statistical significance of difference in estimated date of divergence of sub-haplogoups between pairs of QGP subpopulation groups was calculated using Wilcoxon Rank Sum/Mann Whitney Test.

### Variant annotation

Variants were annotated using dbSNP build 151 (http://www.ncbi.nlm.nih.gov/SNP). Imputed variants were annotated using Variant Effect Predictor^79^ version 84 which uses Ensembl database v84^80^ and Genecode v19 on GRCh37^81^.

### Haplotype phasing and reference panel generation

Processed QGP phase 1 dataset (Where we excluded SNVs with missingness rate of >1% and Hardy–Weinberg equilibrium test *P* value < 10^−5^) was phased using Eagle (v2.3.5) with reference-independent parameter^82^. For that, bcftools missing2ref plugin was used to annotate missing sites to the reference allele and multiallelic sites were split, as required by the tool. Phasing accuracy was defined as the number of switch errors present in the phased data set. The phased haplotypes were then converted to Minimac 3 format^59^.

### Genotype imputation

Imputation using the various reference panels were performed on a High-Performance Computing Cluster at Sidra Medicine using Minimac3^59^ with default parameters. High-confidence imputed variants were defined as having Minimac 3 r^2^ score >0.5.

### Imputation accuracy

We used measureAggregateRsquared developed at Welcome Trust Center for Human Genetics (https://github.com/winni2k/measureAggregateRsquared) to calculate concordance between imputed and observed genotypes in order to assess performance of imputation using various combinations of arrays and imputation panels. Comparisons were performed for five allele frequency bins defined with the thresholds (0.001, 0.01, 0.1, 0.5, 1). Also, comparisons were performed by further filtering on Minimac3 r^2^ score.

### Reporting summary

Further information on research design is available in the Nature Research Reporting Summary linked to this article.

## Supplementary information

Supplementary Information

Description of Additional Supplementary Files

Supplementary Data 1

Reporting Summary

## Data Availability

Access to the genotypic data used for this study and the imputation panel is through a dedicated portal by QGP (Accession ID: QF-QGP-RES-PUB-007). The informed consent given by the study participants does not cover posting of participant level phenotype and genotype data of Qatar Biobank (QBB)/Qatar Genome Project (QGP) in public databases. Access to QBB/QGP data can be obtained through an established ISO-certified process by submitting a project request at https://www.qatarbiobank.org.qa/research/how-apply which is subject to approval by the QBB IRB committee. Other datasets used in this study are from 1000 Genomes Project (ftp://ftp.1000genomes.ebi.ac.uk/vol1/ftp/), The Human Origin project (https://reich.hms.harvard.edu/datasets), the Greater Middle East study (GME, *n* = 731) (http://www.ncbi.nlm.nih.gov/projects/gap/cgi-bin/study.cgi?study_id=phs000288.v1.p1) as well as previously published whole genomes of Qataris (*n* = 105) (http://www.ncbi.nlm.nih.gov/Traces/study/?acc=SRP060765%2CSRP061943%2CSRP061463&go=go). Source data are provided with this paper.

## Source data

Source Data

## Code availability

The study utilized previously published analysis tools as described in the methods section. No custom tools were used in this study.
