# Supplementary material for: Thousands of Qatari genomes inform human migration history and improve imputation of Arab haplotypes
Source: Nat Commun. 2021 Oct 12;12:5929. doi: 10.1038/s41467-021-25287-y (PMC8511259; doi:10.1038/s41467-021-25287-y)
Supplement: Supplementary file 1 — Supplementary Information [file 41467_2021_25287_MOESM1_ESM.pdf]

Supplementary information

Thousands of Qatari genomes inform human migration history and improve imputation of Arab haplotypes

Rozaimi Mohamad Razali[1], Juan Rodriguez-Flores[2], Mohammadmersad Ghorbani[1], Haroon Naeem[1], Waleed Aamer[1], Elbay Aliyev[1], Ali Jubran[1], Qatar Genome Program Research Consortium[+], Andrew G. Clark[3], Khalid Fakhro[1,4,5,*], Younes Mokrab[1,4,5,*]

1 Department of Human Genetics, Sidra Medicine, Doha, Qatar

2 Department of Genetic Medicine, Weill Cornell Medicine, New York, U.S.A.

3 Department of Molecular Biology and Genetics, Cornell University, New York, U. S. A.

4 Weill Cornell Medicine-Qatar, Doha, Qatar

5 College of Health and Life Sciences, Hamad Bin Khalifa University, Doha, Qatar

+ List of consortium authors and their affiliations appears at the end of the paper.

* Corresponding authors. Emails: ymokrab@sidra.org and kfakhro@sidra.org

Chromosomes	SNVs	Indels
1	5,713,943	498,959
2	6,261,812	522,101
3	5,257,651	433,266
4	5,089,558	429,915
5	4,713,582	391,367
6	4,398,286	383,335
7	4,137,020	363,019
8	4,119,566	315,951
9	3,132,653	265,320
10	3,502,002	296,646
11	3,546,168	289,565
12	3,460,852	303,119
13	2,531,432	219,602
14	2,344,418	203,550
15	2,073,845	185,056
16	2,382,739	188,630
17	2,052,886	193,293
18	2,019,571	171,221
19	1,613,808	161,854
20	1,632,340	134,953
21	956,444	85,603
22	967,656	88,965
X	2,820,409	417,042
Y	54,585	17,806
**Total**	**74,783,226**	**6,560,138**

Supplementary Table 1. High quality variants called in 6,218 samples from the QGP dataset. Abbreviation: SNVs stands for Single Nucleotide Variants. Numbers are prior filtering for HWE, MAF and missingness.

Superpopulation code	Superpopulation name	Subpopulation code	Subpopulation name
AFR	African	ACB	African Caribbean in Barbados
		ASW	African Ancestry in Southwest US
		GWD	Gambian in Western Division, The Gambia - Mandinka
		MSL	Mende in Sierra Leone
		ESN	Esan in Nigeria
		YRI	Yoruba in Ibadan, Nigeria
		LWK	Luhya in Webuye, Kenya
AMR	American	PUR	Puerto Rican in Puerto Rico
		CLM	Colombian in Medellin, Colombia
		PEL	Peruvian in Lima, Peru
		MXL	Mexican Ancestry in Los Angeles, California
EAS	East Asian	CHS	Han Chinese South
		KHV	Kinh in Ho Chi Minh City, Vietnam
		CHB	Han Chinese in Beijing, China
		JPT	Japanese in Tokyo, Japan
		CDX	Chinese Dai in Xishuangbanna, China
EUR	European	FIN	Finnish in Finland
		TSI	Toscani in Italy
		IBS	Iberian populations in Spain
		CEU	Utah residents (CEPH) with Northern and Western European ancestry
		GBR	British in England and Scotland
SAS	South Asian	BEB	Bengali in Bangladesh
		PJL	Punjabi in Lahore, Pakistan
		GIH	Gujarati Indians in Houston, TX
		ITU	Indian Telugu in the UK
		STU	Sri Lankan Tamil in the UK

Supplementary Table 2. Abbreviations of 1KG populations.

Panel	QGP	1000G	CAAPA	HAPMAP2	HRC
**Geographical location**	Middle east (Qatar)	26 cohorts worldwide	19 cohorts (African Americans)	4 cohorts (Africa, Europe, China, Japan)	20 cohorts mostly European ancestry
**Average sequencing coverage**	30x	7.4x	30x	-	4-8x
**Sequencing method**	WGS	WGS, WES	WGS	Array	WGS and WES
**Number of haplotypes**	12,432	5,008	1,766	540	64,976
**Number of autosomal SNVs**	68,107,887	81,027,987	31,163,897	2,542,916	39,235,157

Supplementary Table 3. Details of reference panels compared against in this study. Abbreviations: WGS, Whole Genome Sequencing; WES, Whole Exome Sequencing.

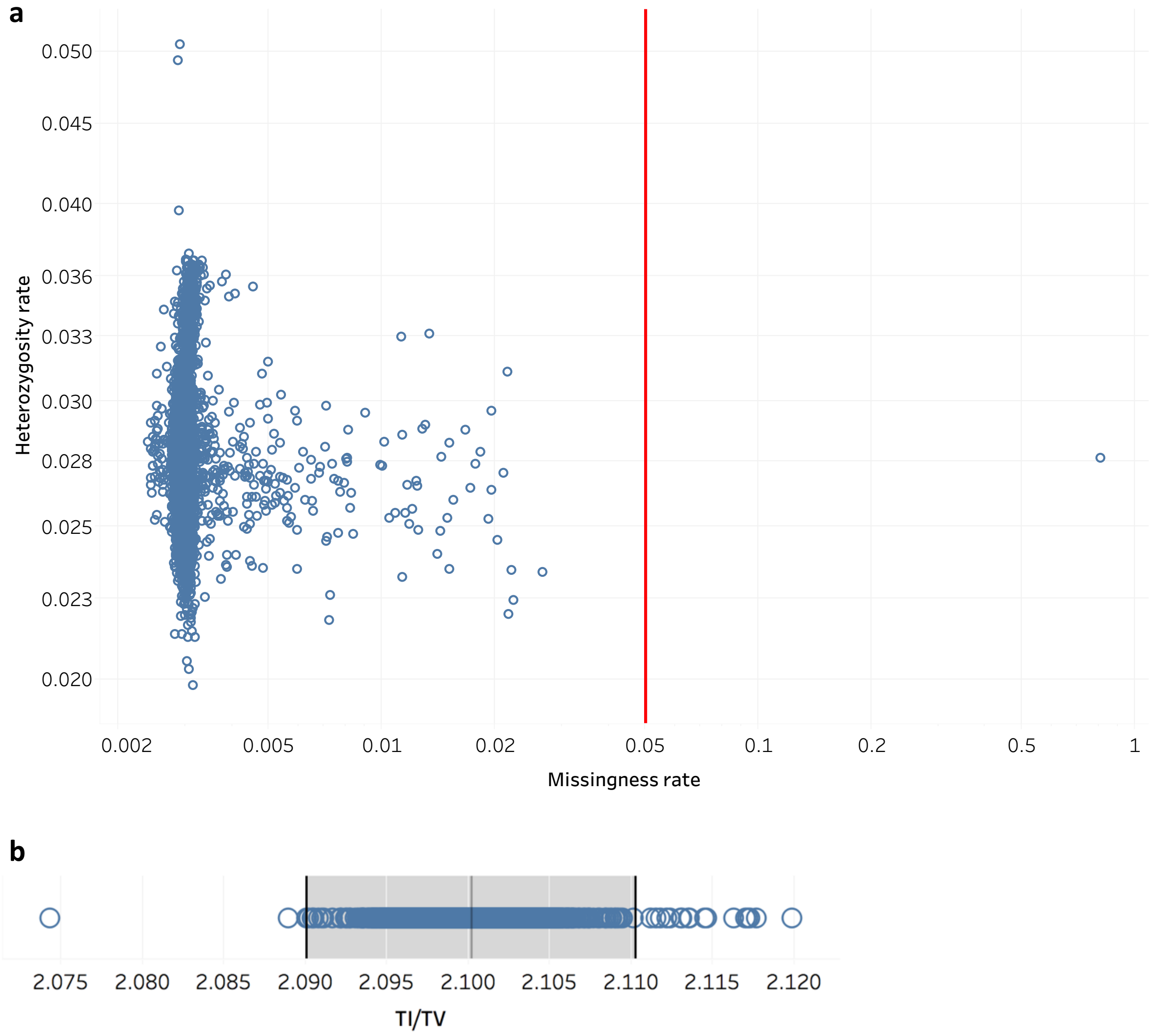

Supplementary Fig.1. Heterozygosity and transition/transversion rate of the study samples. (a) Heterozygosity rate against missingness rate for all individuals prior to QC. One sample shows overall high missingness (0.81) thus was excluded from downstream study. A second sample was removed due to high missingness in chromosome 18 **(b)** Distribution of TI/TV for all samples (6,218) prior to QC. Box indicate +/-4 SDEV around the mean. All samples are within the expected range for human (~ 2).

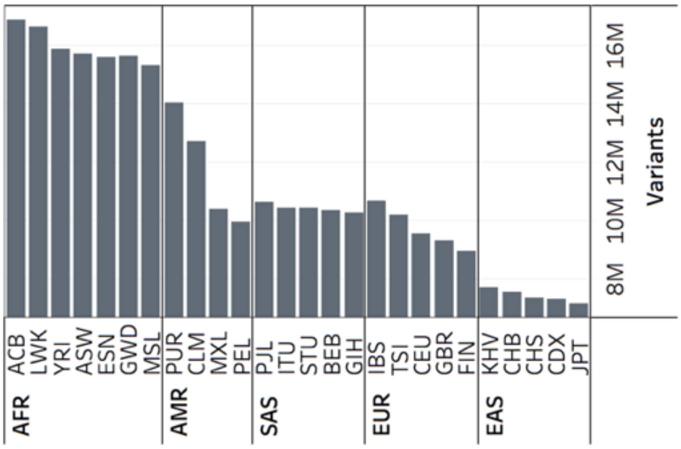

Supplementary Fig.2. Allele sharing between QGP and 26 world populations form 1KG phase 3. Abbreviations are explained in **Abbreviations** section**.**

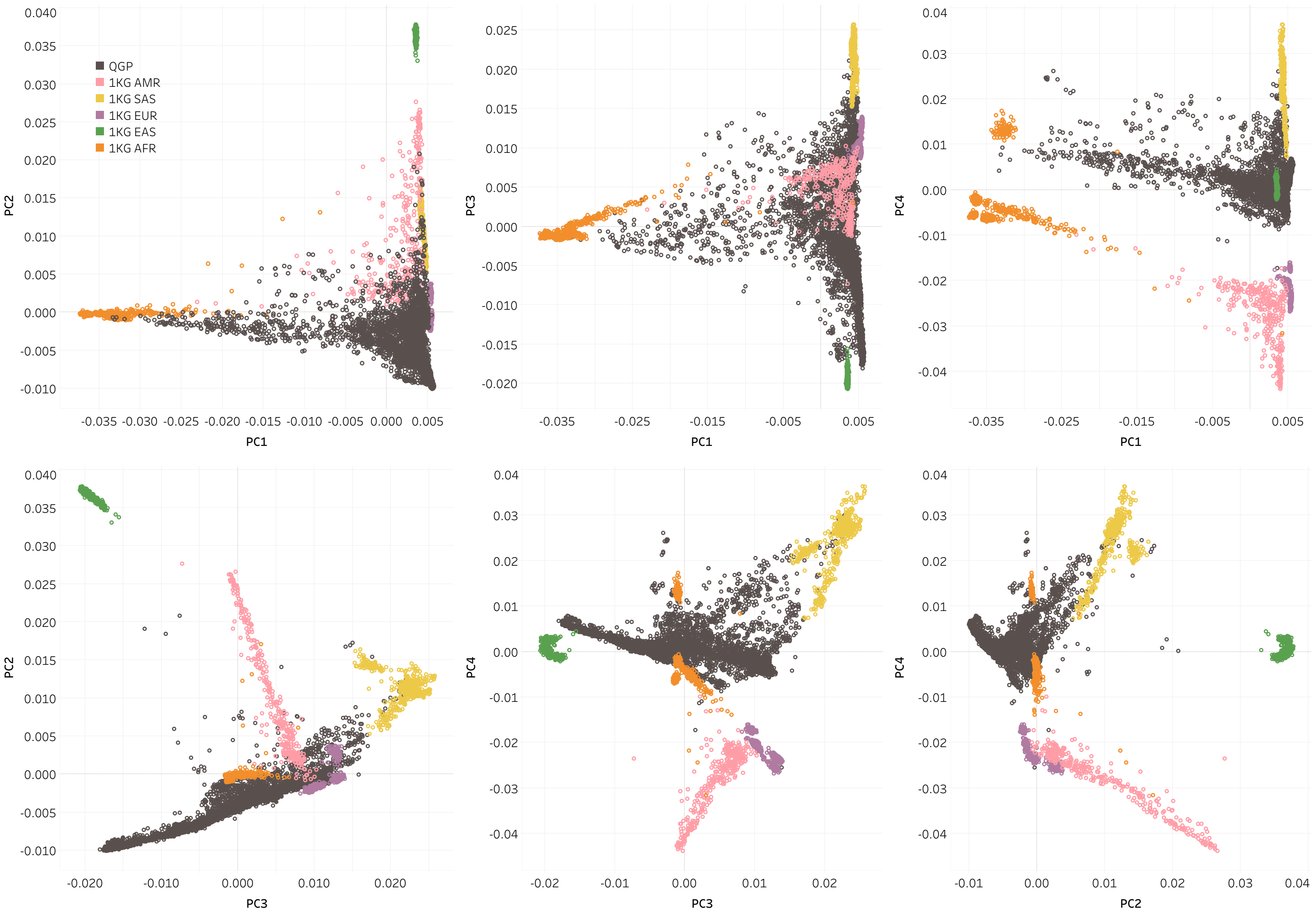

Supplementary Fig.3. PCA of QGP and 1KG populations. Pairwise Plots of principal components (PC1-PC4) highlighting in different colors the QGP dataset (black) and the five reference superpopulations from 1KG Project phase 3 (other colors).

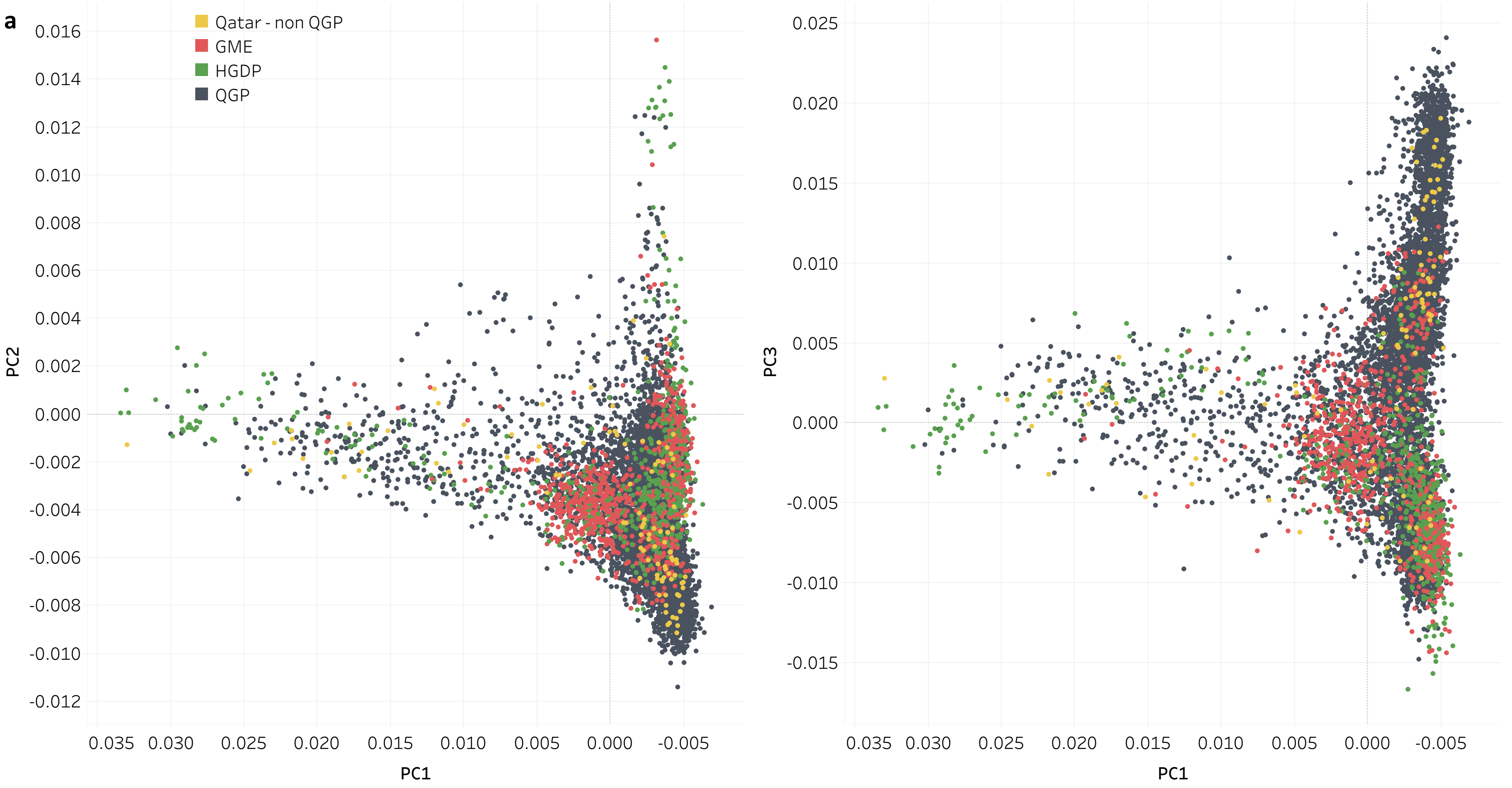

Supplementary Fig.4. P1-PC2 and PC1-PC3 plots of the combined dataset of QGP and diverse populations from Human Origin (HO) and GME datasets and other publicly available Qatari samples. Samples are colored by dataset on a background of QGP samples shown in black.

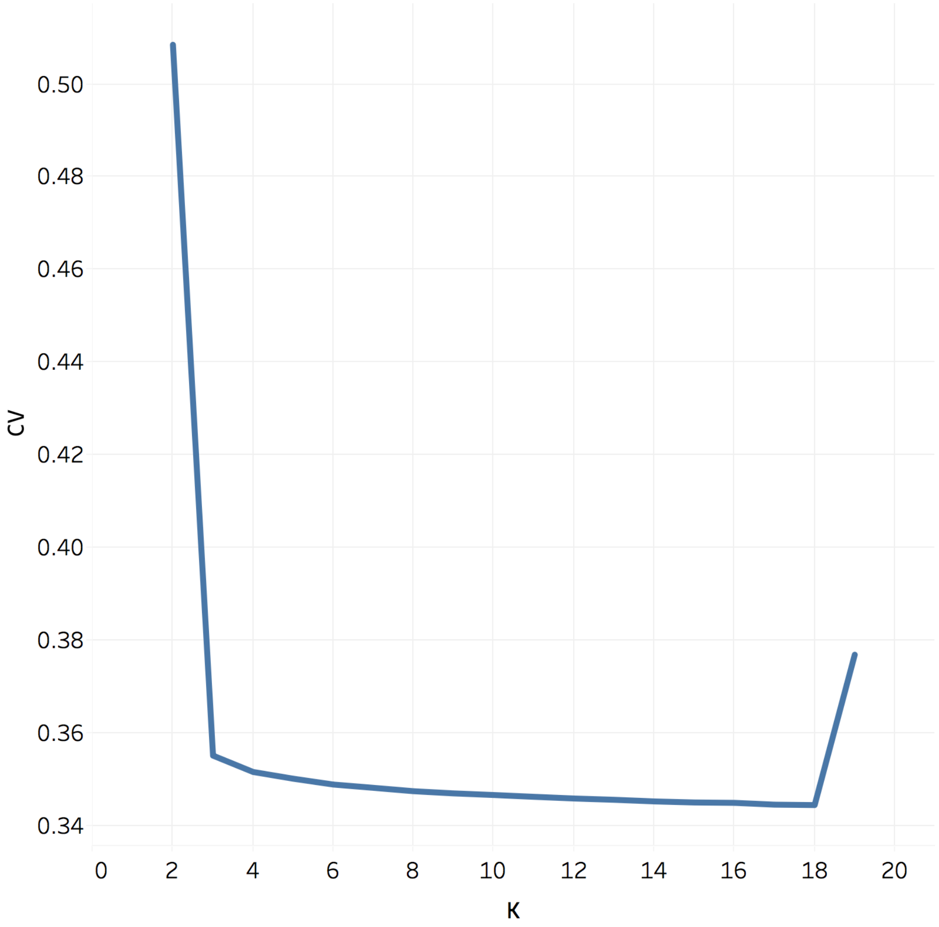

Supplementary Fig.5. Cross validation error for population structure analysis using ADMIXTURE. 5-fold cross validation was performed for each *k* value from 2 to 19 showing a plateau of minimal error values between *k*=8 and *k*=18. Grey box shows median and 99% confidence intervals.

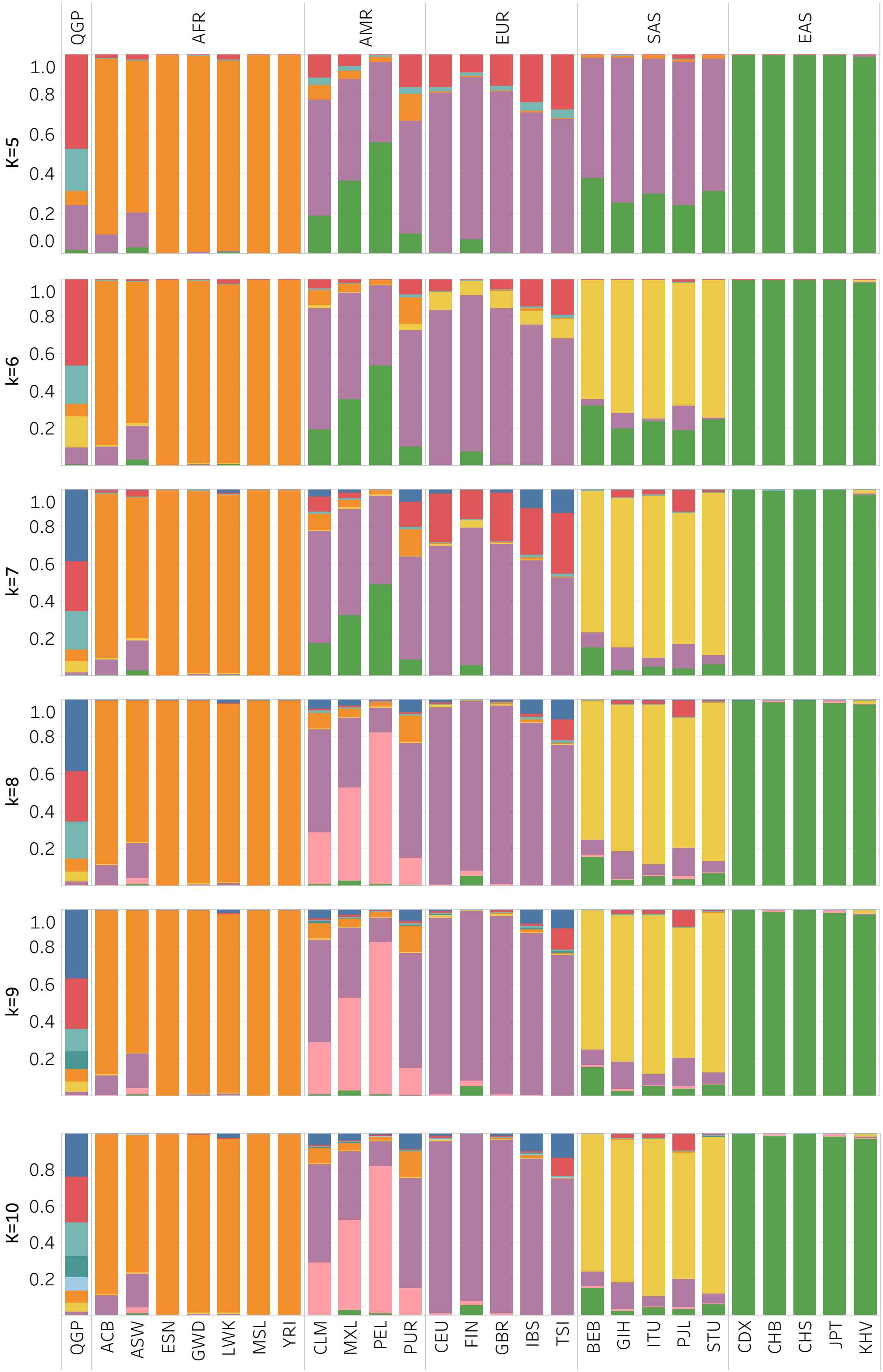

Supplementary Fig.6. Average ancestral fractions for QGP and 1KG populations. Results are shown for *k* [5-10] from the analysis with ADMIXTURE.

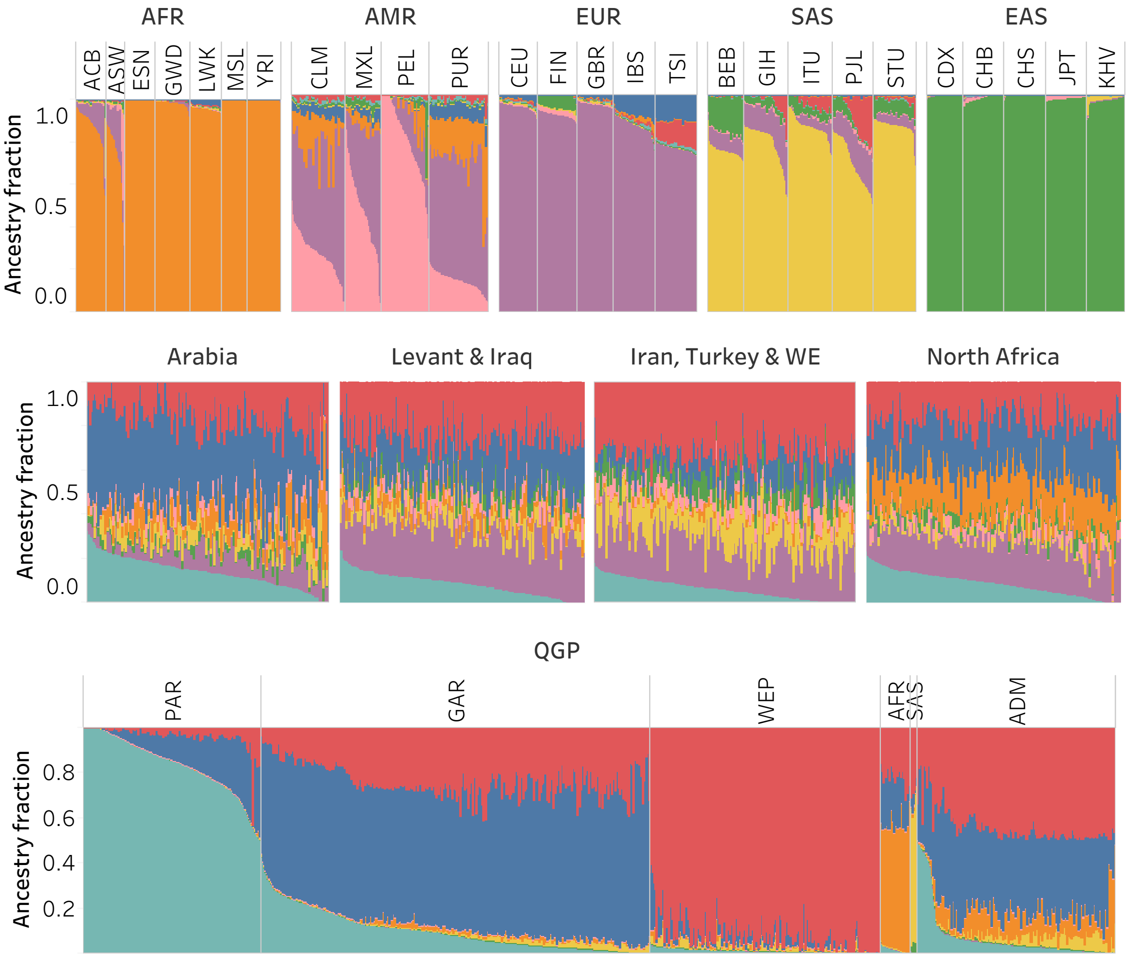

Supplementary Fig. 7. Global ancestry fractions inferred from ADMIXTURE on (top) 1KG populations, (Middle) Human Origin and GME cohorts and (bottom) QGP samples. *k*=8. QGP samples are grouped based on major ancestry. PAR, GAR, WEP, AFR, SAS and ADM are labels assigned based on major ancestry.

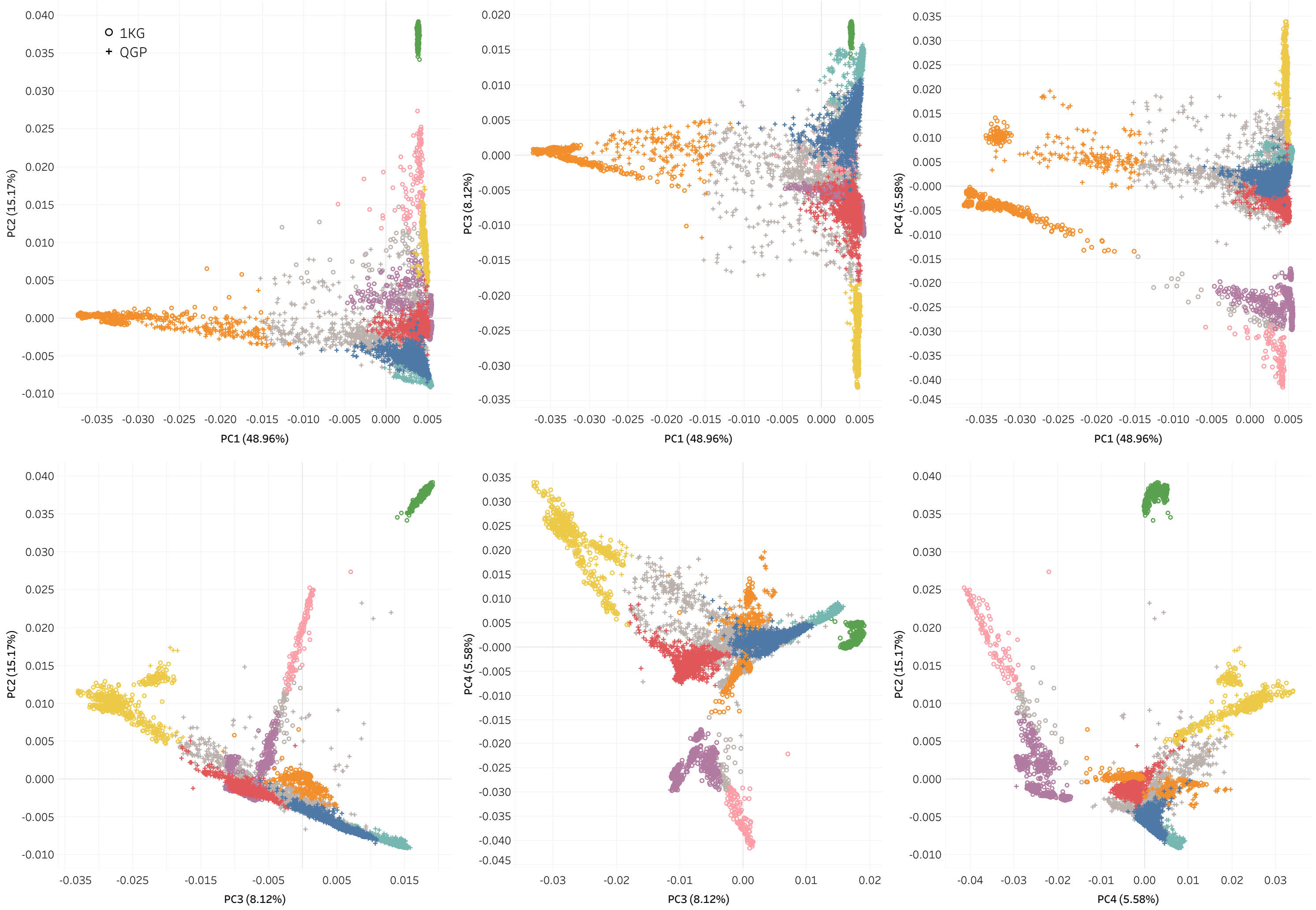

Supplementary Fig.8. PCA colored by major ancestries. PCA plots of QGP and 1KG colored based on major ancestries from ADMIXTURE. QGP dataset is indicated by plus sign and 1KG by circle. Admixed individuals are shown in grey.

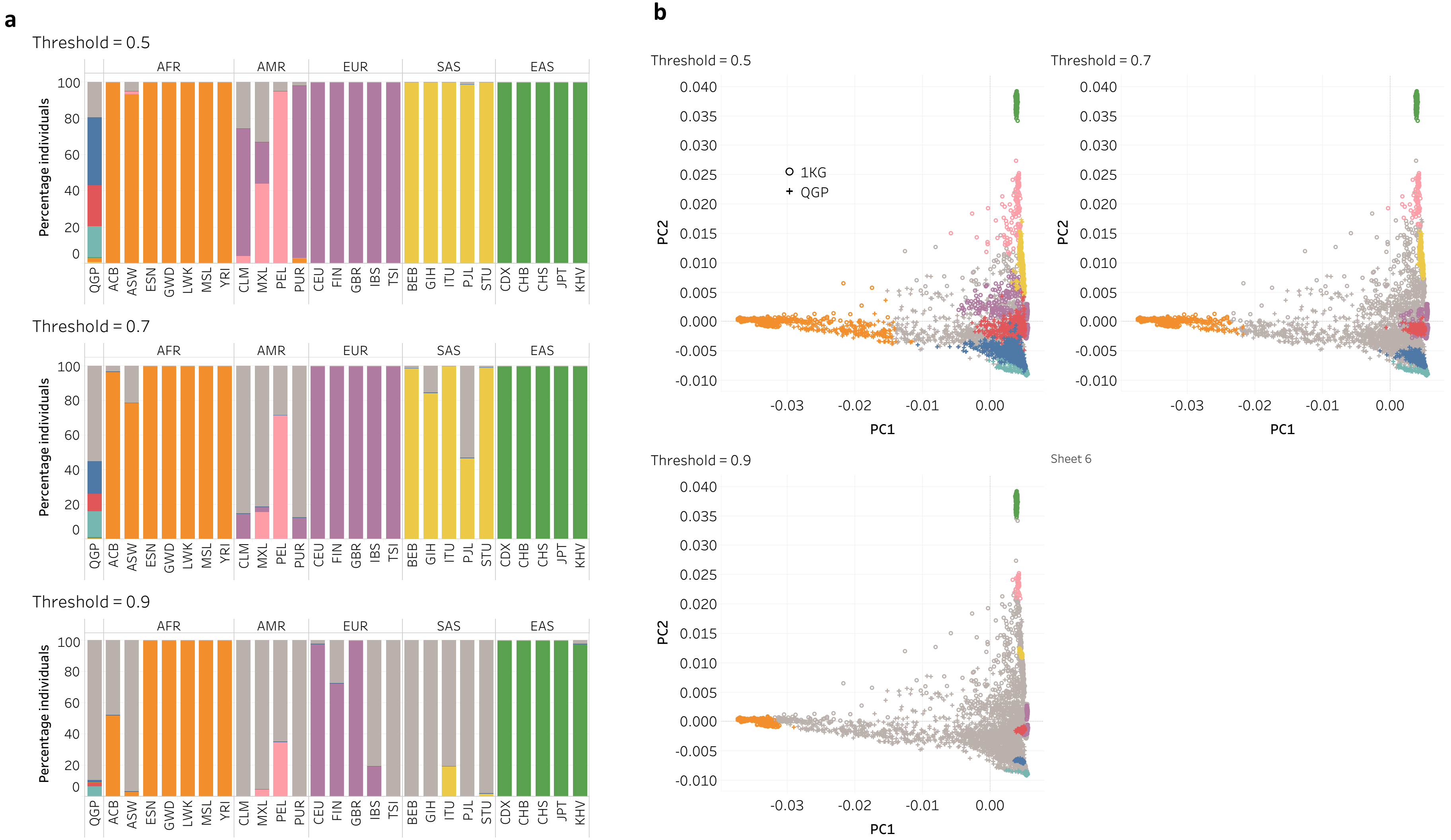

.

Supplementary Fig. 9. Effect of varying the threshold for assigning a dominant ADMIXTURE ancestry fraction to the QGP and 1KG samples. (a) Percentage of samples assigned to each of the eight ancestries obtained from ADMIXTURE (using *k*=8) based on the largest ancestral fraction greater than 0.5, 0.7 and 0.9 respectively. Samples with no assignable dominant ancestry using each threshold are shown in grey. **(b)** PCA plots showing samples colored by dominant ancestries defined with the respective thresholds.

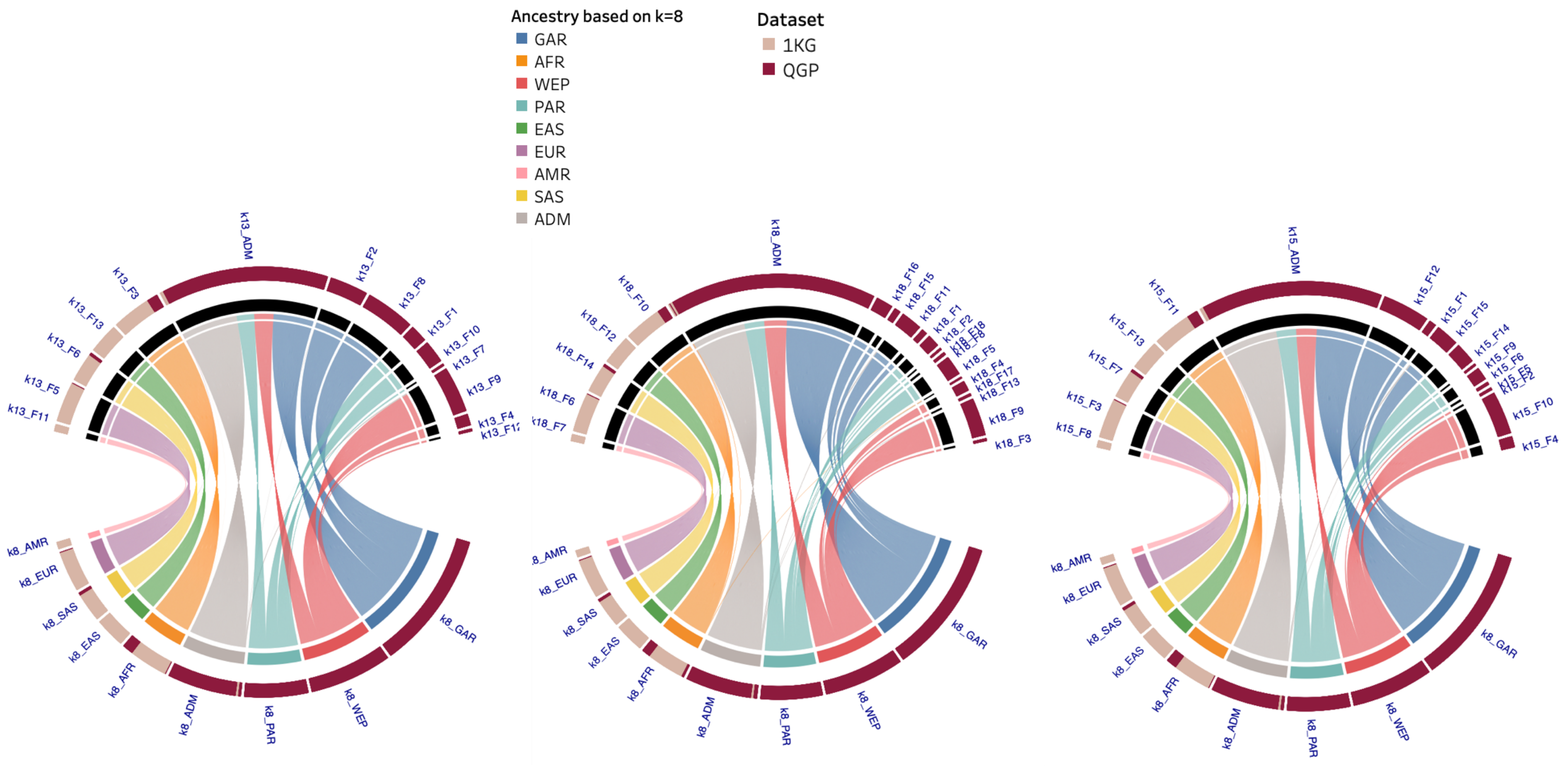

***k*=8 vs. *k*=13** ***k*=8 vs. *k*=15** ***k*=8 vs. *k*=18**

Supplementary Figure 10. Mapping of samples between genetic clusters generated based on ADMIXTURE (*k*=8) and those based on *k*=13, 15 and 18.

a

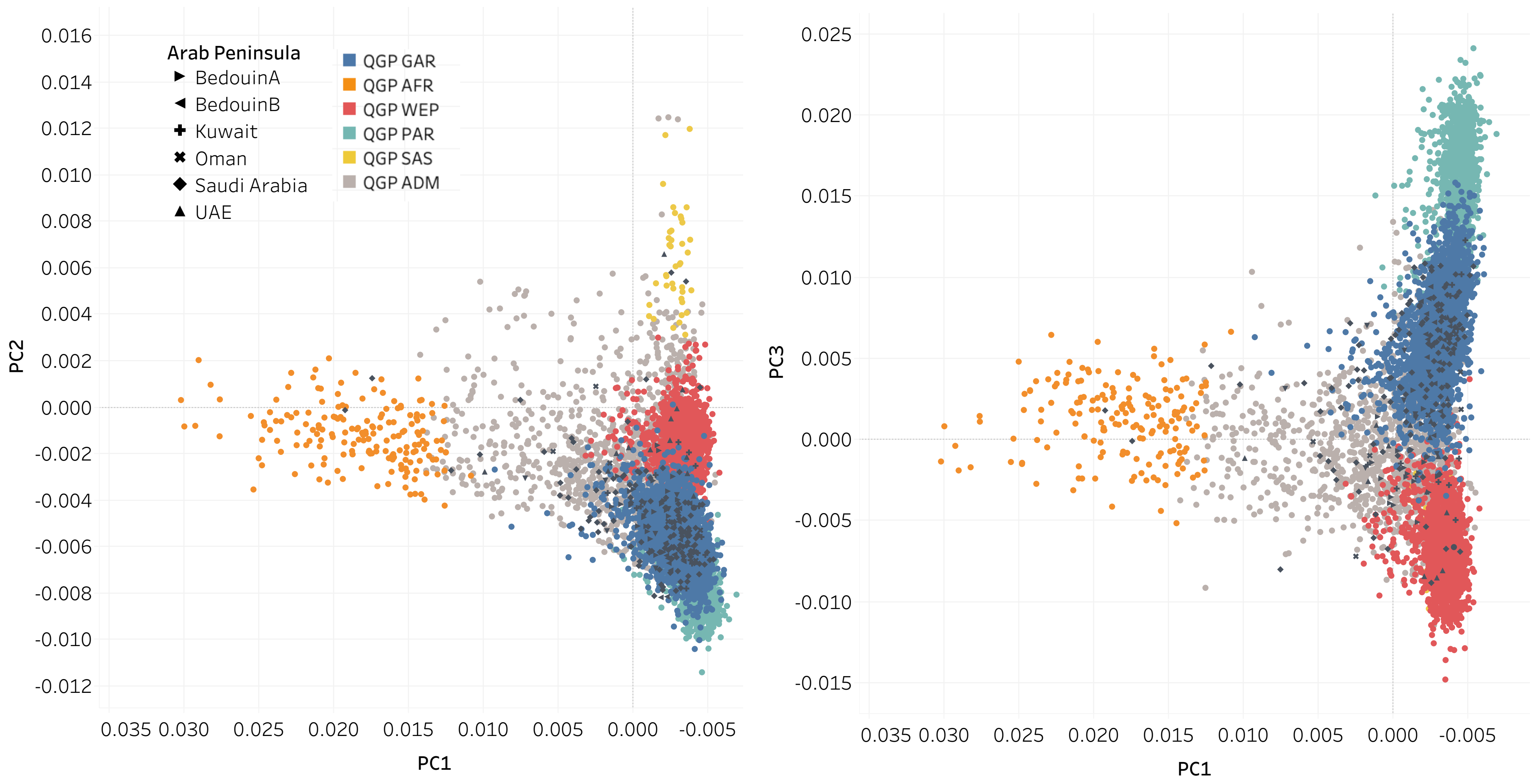

Supplementary Fig. 11. PCA plots of QGP dataset and diverse Middle Eastern populations from Human Origin (HO), GME datasets and other publicly available Qatari samples. QGP genetic clusters are shown in color. Black shapes depict subjects from the regions of **(a)** Arab Peninsula, **(b)** other non-QGP Qataris, **(c)** Levant and Iraq, **(d)** North Africa, **(e)** Iran, Turkey and West Eurasia, **(f)** East Africa and **(g)** Jews.

b

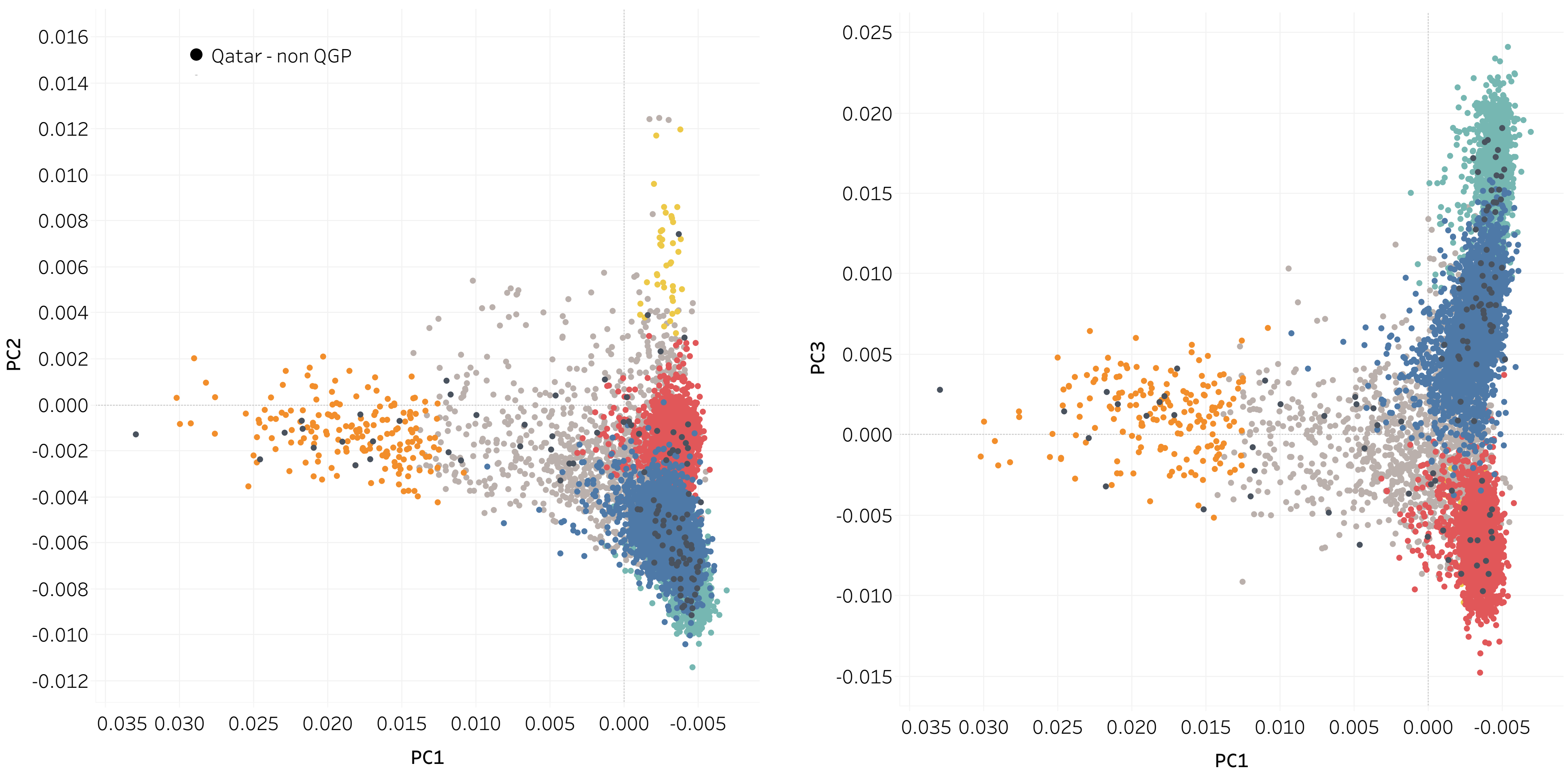
Qatar - non QGP
0.016
0.014
0.012
0.010
0.008
0.006
0.004
0.002
0.000
-0.002
-0.004
-0.006
-0.008
-0.010
-0.012
PC2
0.035 0.030 0.025 0.020 0.015 0.010 0.005 0.000 -0.005
PC1
0.025
0.020
0.015
0.010
0.005
0.000
-0.005
-0.010
-0.015
PC3
0.035 0.030 0.025 0.020 0.015 0.010 0.005 0.000 -0.005
PC1

c

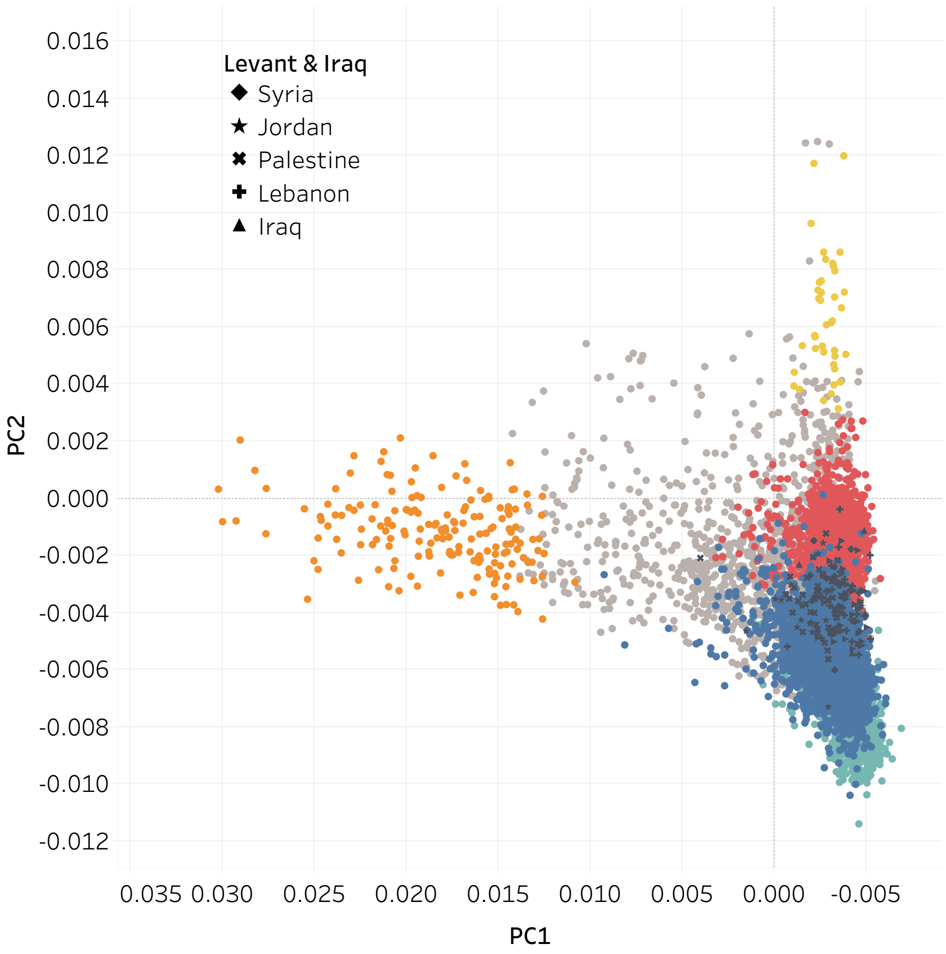
Levant & Iraq
Syria
Jordan
Palestine
Lebanon
Iraq
PC2
0.016
0.014
0.012
0.010
0.008
0.006
0.004
0.002
0.000
-0.002
-0.004
-0.006
-0.008
-0.010
-0.012
0.035
0.030
0.025
0.020
0.015
0.010
0.005
0.000
-0.005
PC1

d

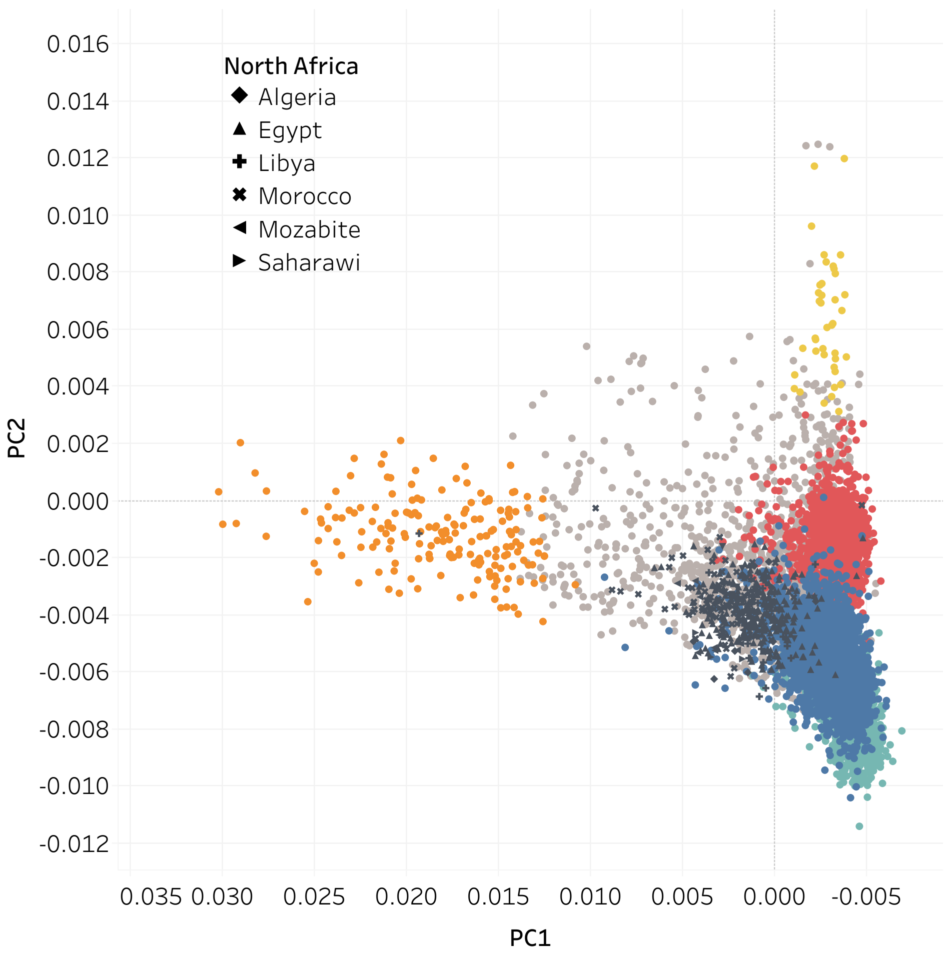
North Africa
Algeria
Egypt
Libya
Morocco
Mozabite
Saharawi
PC2
0.016
0.014
0.012
0.010
0.008
0.006
0.004
0.002
0.000
-0.002
-0.004
-0.006
-0.008
-0.010
-0.012
0.035
0.030
0.025
0.020
0.015
0.010
0.005
0.000
-0.005
PC1

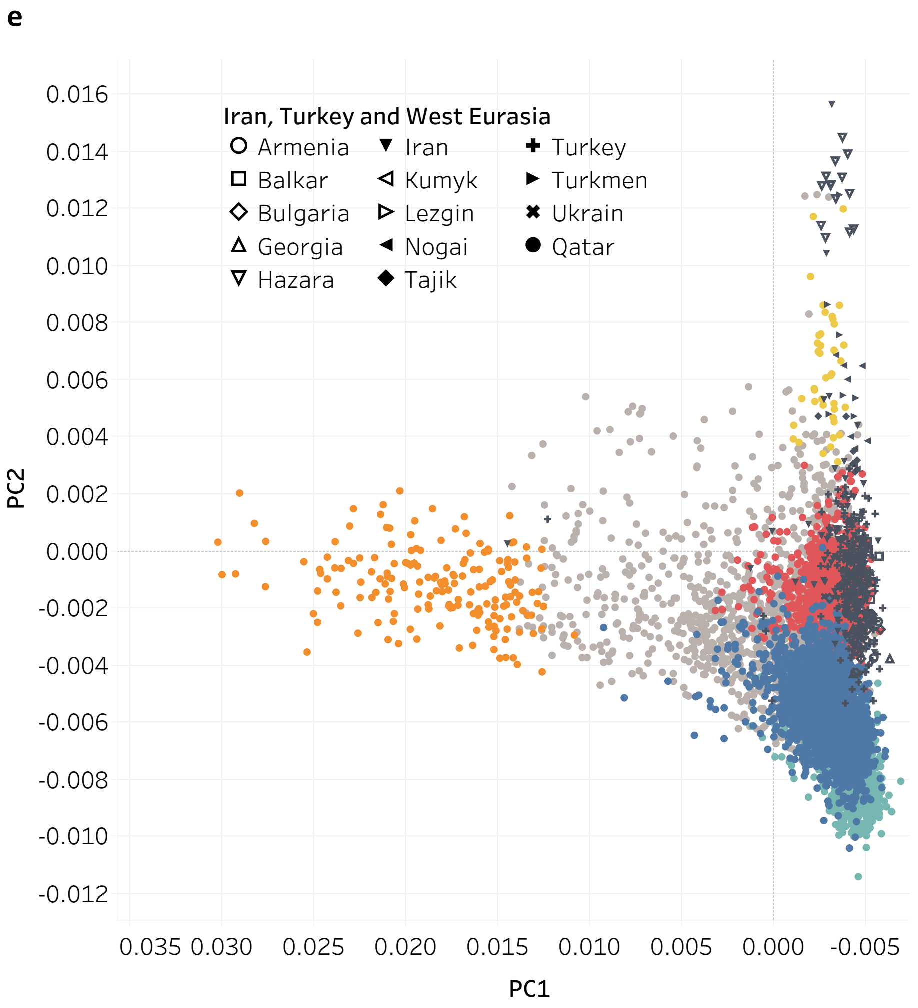
e
0.016
0.014
0.012
0.010
0.008
0.006
0.004
0.002
0.000
-0.002
-0.004
-0.006
-0.008
-0.010
-0.012
PC2
Iran, Turkey and West Eurasia
Armenia
Balkar
Bulgaria
Georgia
Hazara
Iran
Kumyk
Lezgin
Nogai
Tajik
Turkey
Turkmen
Ukrain
Qatar
0.035 0.030 0.025 0.020 0.015 0.010 0.005 0.000 -0.005
PC1

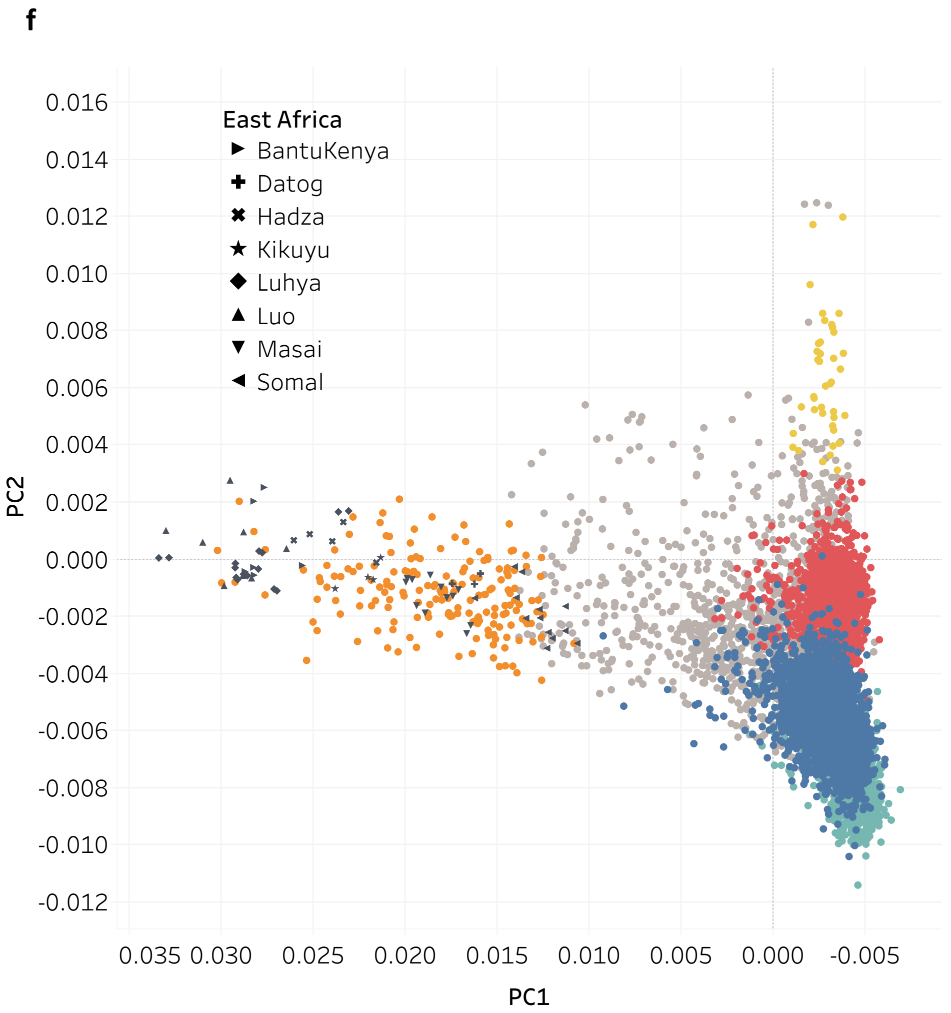
f
0.016
0.014
0.012
0.010
0.008
0.006
0.004
0.002
0.000
-0.002
-0.004
-0.006
-0.008
-0.010
-0.012
PC2
East Africa
BantuKenya
Datog
Hadza
Kikuyu
Luhya
Luo
Masai
Somal
0.035 0.030 0.025 0.020 0.015 0.010 0.005 0.000 -0.005
PC1

g

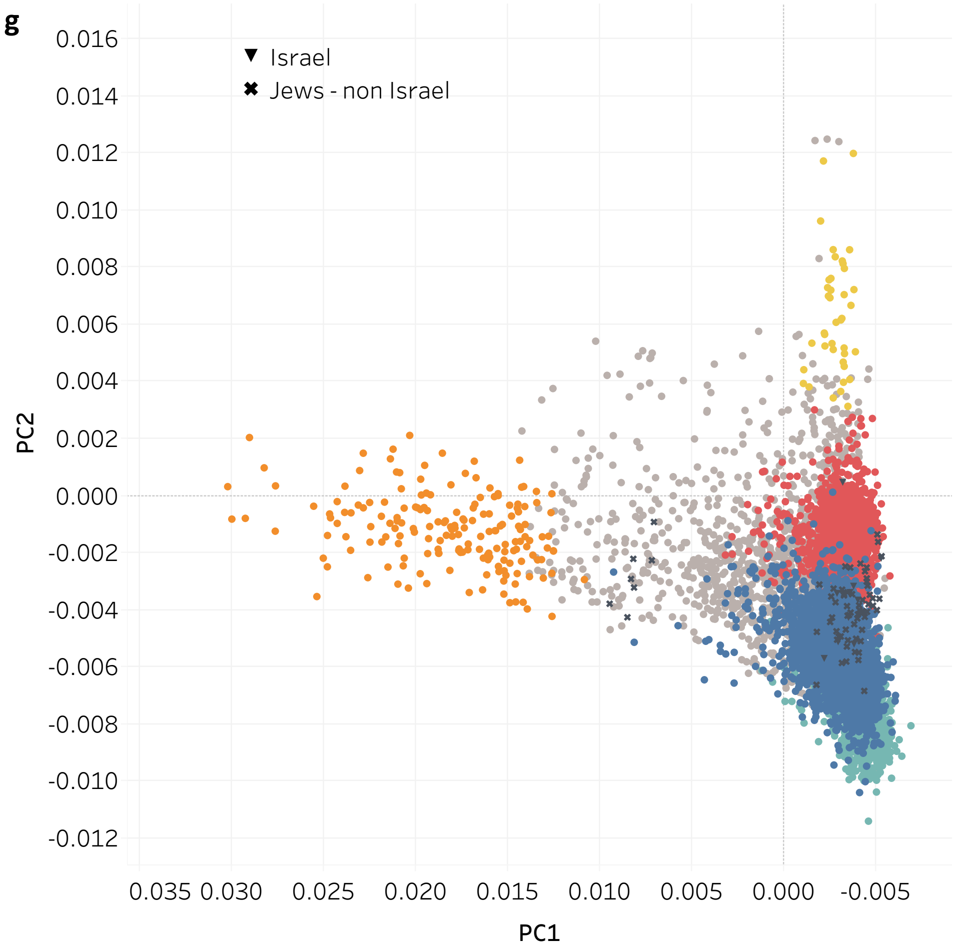

Israel
Jews - non Israel
0.016
0.014
0.012
0.010
0.008
0.006
0.004
0.002
0.000
-0.002
-0.004
-0.006
-0.008
-0.010
-0.012
PC2
0.035
0.030
0.025
0.020
0.015
0.010
0.005
0.000
-0.005
PC1

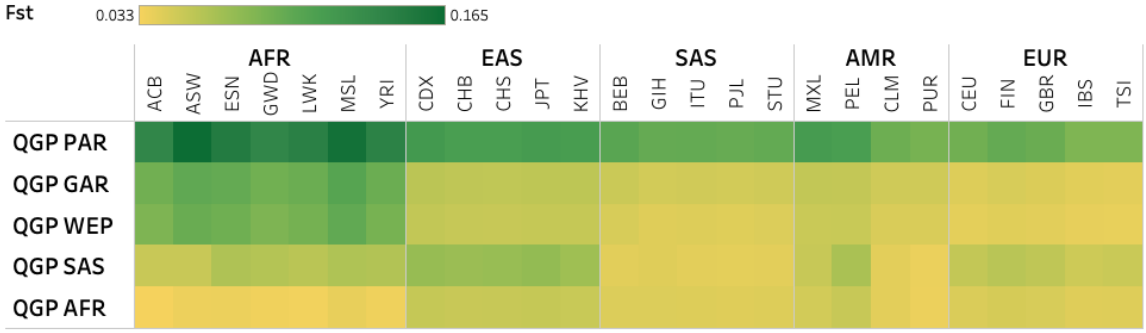

Supplementary Fig. 12. Genetic distance between QGP and 1KG populations inferred from F_{ST} values between QGP sub-populations and the 26 reference populations from 1KG.

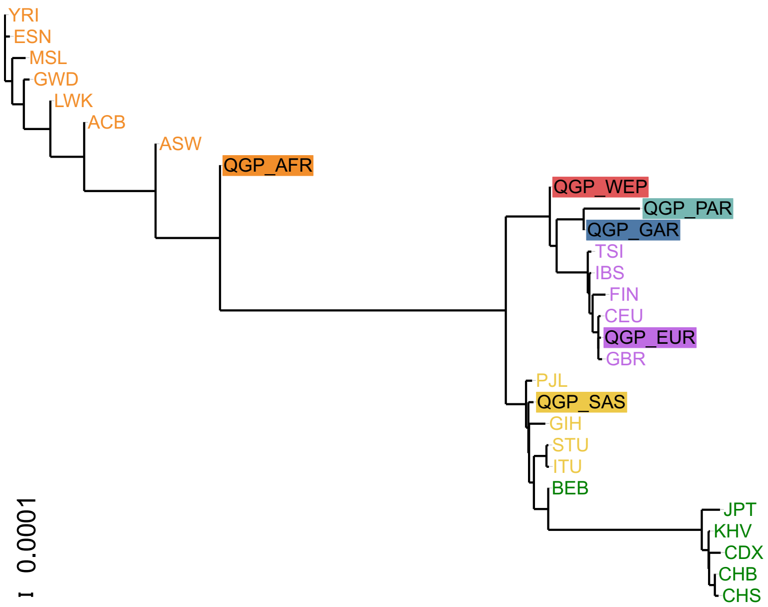

Supplementary Fig. 13. TreeMix-based tree of QGP and 1KG populations showing divergence patterns based on genetic drift.

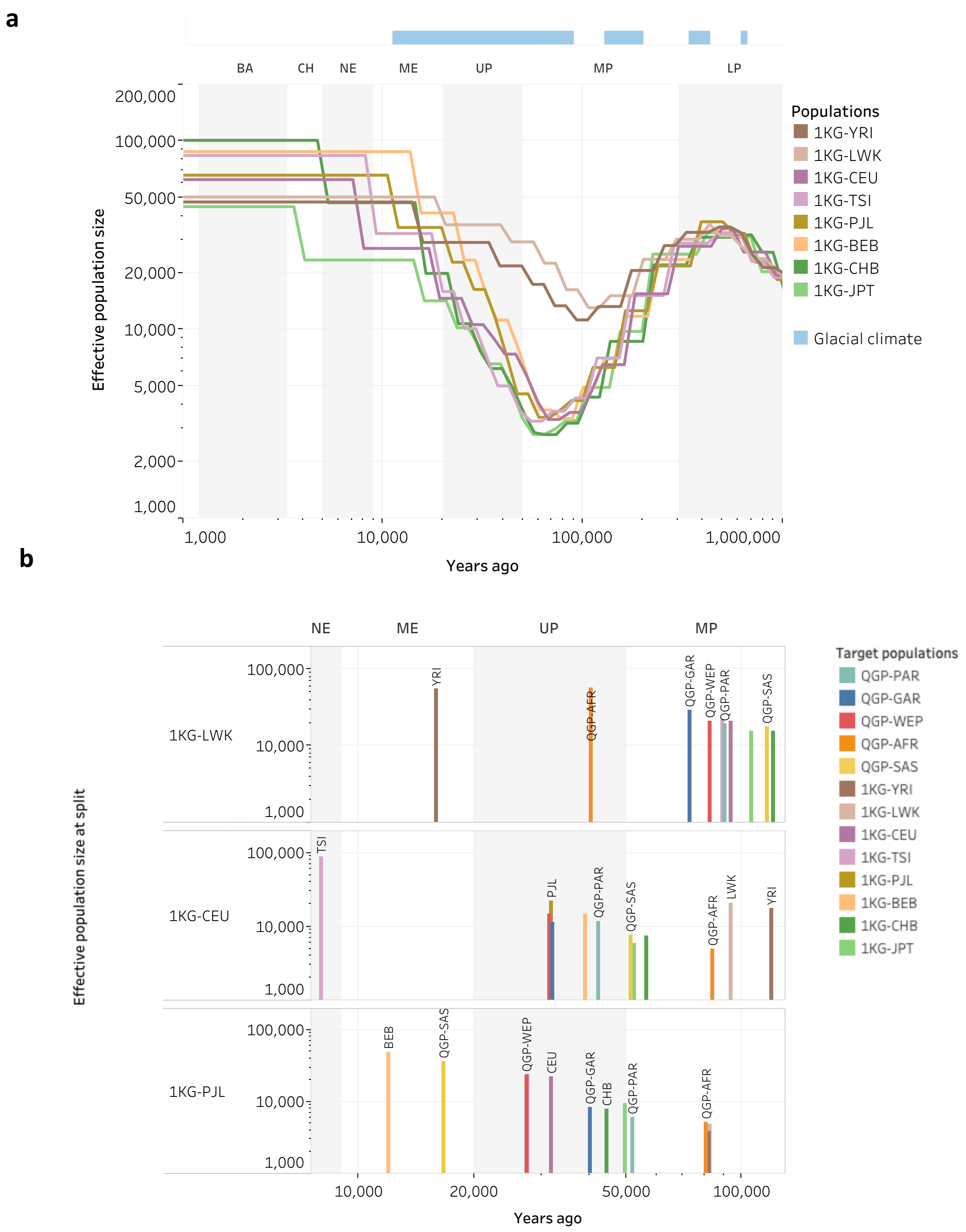

Supplementary Fig. 14. Divergence of the Qatari sub-populations relative to world populations. (a) Effective population size over time for representative populations from Africa, Europe and South Asia, inferred using SMC++. **(b)** Estimated split times for representative continental populations relative to other Qatari and world populations**.** Archeological periods are highlighted with alternating grey and white backgrounds and labeled as LP (Lower Paleolithic), MP (Middle Paleolithic), UP (Upper Paleolithic), ME (Mesolithic), Neolithic (NE), Chalcolithic (CL), Bronze Age (BA). Glacial periods are indicated horizontally on the top.

Age	Max. Early BCE	Min. Late BCE	Country	Label	Distinct count of Sample ID	Dataset
Upper Paleolithic	45,530	40,610	Russia	Ust_Ishim	1	Lazaridis et al. 2016
	36,730	34,310	Russia	Kostenki14	1	Lazaridis et al. 2016
	22,570	22,140	Russia	MA1	1	Lazaridis et al. 2016
Mesolithic	11,840	9,760	Israel	Natufian	6	Lazaridis et al. 2016
	11,820	11,610	Switzerland	Switzerland..	1	Lazaridis et al. 2016
	11,430	7,600	Georgia	CHG	2	Lazaridis et al. 2016
	9,100	8,600	Iran	Iran_HotuIIIb	1	Lazaridis et al. 2016
Neolithic	8,500	Null	USA	Kennewick	1	Lazaridis et al. 2016
	8,300	6,700	Jordan	Levant_N	12	Lazaridis et al. 2016
	8,202	7,613	Iran	Iran_N	5	Lazaridis et al. 2016
	7,300	6,750	Israel	Levant_N	1	Lazaridis et al. 2016
	6,850	5,000	Russia	EHG	3	Lazaridis et al. 2016
	6,500	5,600	Turkey	Anatolia_N	24	Lazaridis et al. 2016
	6,210	5,990	Luxembourg	WHG	1	Lazaridis et al. 2016
	5,983	5,747	Spain	WHG	1	Lazaridis et al. 2016
	5,964	5,516	Sweden	SHG	6	Lazaridis et al. 2016
	5,837	5,659	Iran	Iran_LN	1	Lazaridis et al. 2016
	5,780	5,640	Hungary	WHG	1	Lazaridis et al. 2016
	5,710	4,360	Hungary	Europe_EN	10	Lazaridis et al. 2016
	5,500	4,775	Germany	Europe_EN	14	Lazaridis et al. 2016
	5,469	5,066	Spain	Europe_EN	5	Lazaridis et al. 2016
Chalcholitic	5,200	4,000	Russia	Steppe_Ene..	3	Lazaridis et al. 2016
	4,839	3,796	Iran	Iran_ChL	5	Lazaridis et al. 2016
	4,500	Null	Ethiopia	Mota	1	Lazaridis et al. 2016
	4,350	3,700	Armenia	Armenia_ChL	5	Lazaridis et al. 2016
	3,970	3,025	Germany	Europe_MN..	5	Lazaridis et al. 2016
	3,943	3,708	Turkey	Anatolia_ChL	1	Lazaridis et al. 2016
	3,900	2,346	Spain	Europe_MN..	18	Lazaridis et al. 2016
	3,483	1,773	Italy	Europe_MN..	3	Lazaridis et al. 2016
	2,900	2,700	Hungary	Europe_MN..	1	Lazaridis et al. 2016
Bronze age	3,347	2,410	Armenia	Armenia_EB..	3	Lazaridis et al. 2016
	3,339	1,692	Russia	Steppe_EM..	28	Lazaridis et al. 2016
	2,925	1,200	Russia	Steppe_ML..	21	Lazaridis et al. 2016
	2,880	979	Germany	Europe_LN..	43	Lazaridis et al. 2016
	2,851	547	Denmark	Europe_LN..	4	Lazaridis et al. 2016
	2,621	1,132	Sweden	Europe_LN..	6	Lazaridis et al. 2016
	2,500	2,000	Israel	Yehud_IBA	13	Agranat et al. 2020
	2,490	1,966	Jordan	Levant_BA	3	Lazaridis et al. 2016
	2,334	2,149	Israel	Megiddo_IBA	1	Agranat et al. 2020
	2,286	1,693	Poland	Europe_LN..	4	Lazaridis et al. 2016
	2,190	1,110	Hungary	Europe_LN..	12	Lazaridis et al. 2016
	2,120	1,887	Kazakhstan	Steppe_ML..	1	Lazaridis et al. 2016
	1,971	1,278	Israel	Megiddo_M..	30	Agranat et al. 2020
	1,906	855	Armenia	Armenia_ML..	9	Lazaridis et al. 2016
	1,800	1,250	Israel	Hazor_MLBA	3	Agranat et al. 2020
	1,750	1,618	Spain	Iberia_BA	1	Lazaridis et al. 2016
	1,688	1,535	Israel	Megiddo_I1..	1	Agranat et al. 2020
	1,600	1,500	Israel	Megiddo_I2..	1	Agranat et al. 2020
				Megiddo_I2..	1	Agranat et al. 2020
	1,550	1,150	Jordan	Baqah_MLBA	21	Agranat et al. 2020
	1,107	923	Israel	Megiddo_IA	1	Agranat et al. 2020
	Null	Null	Czech Repu..	Europe_LN..	5	Lazaridis et al. 2016
			Estonia	Europe_LN..	1	Lazaridis et al. 2016
Iron age	1,011	846	Israel	Abel_IA	1	Agranat et al. 2020
	375	203	Russia	Steppe_IA	1	Lazaridis et al. 2016

Supplementary Fig. 15. Source and details of the compiled dataset of published ancient human genomes used in the current study.

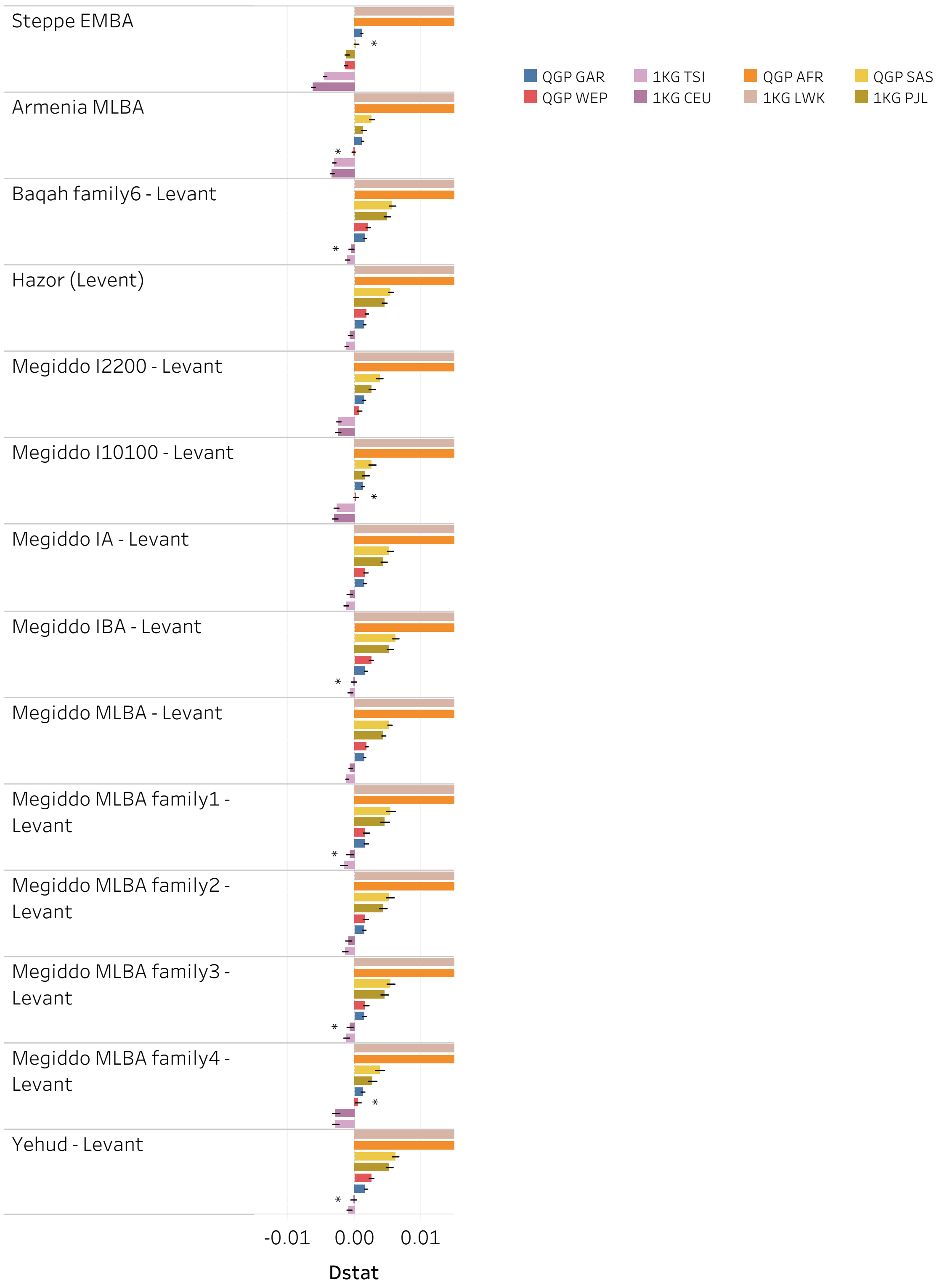

Supplementary Figure 16. Bar plots showing D-statistic for admixture of Peninsular Arabs with other Bronze Age genomes from levant and Stepp regions, relative to QGP and other world populations, inferred from Patterson's D-statistic (D-stat). D-statistics values with low absolute Z score (< 3) are highlighted with *. Black lines at the end of bars indicate 95% confidence intervals. Negative D-statistic value imply higher introgression with Peninsular Arabs relative to other tested populations while positive values imply the opposite. For clarity, bars for QGP AFR and 1KG LWK are shown up to 0.01 (They extend to maxima of 0.03 and 0.05 respectively).

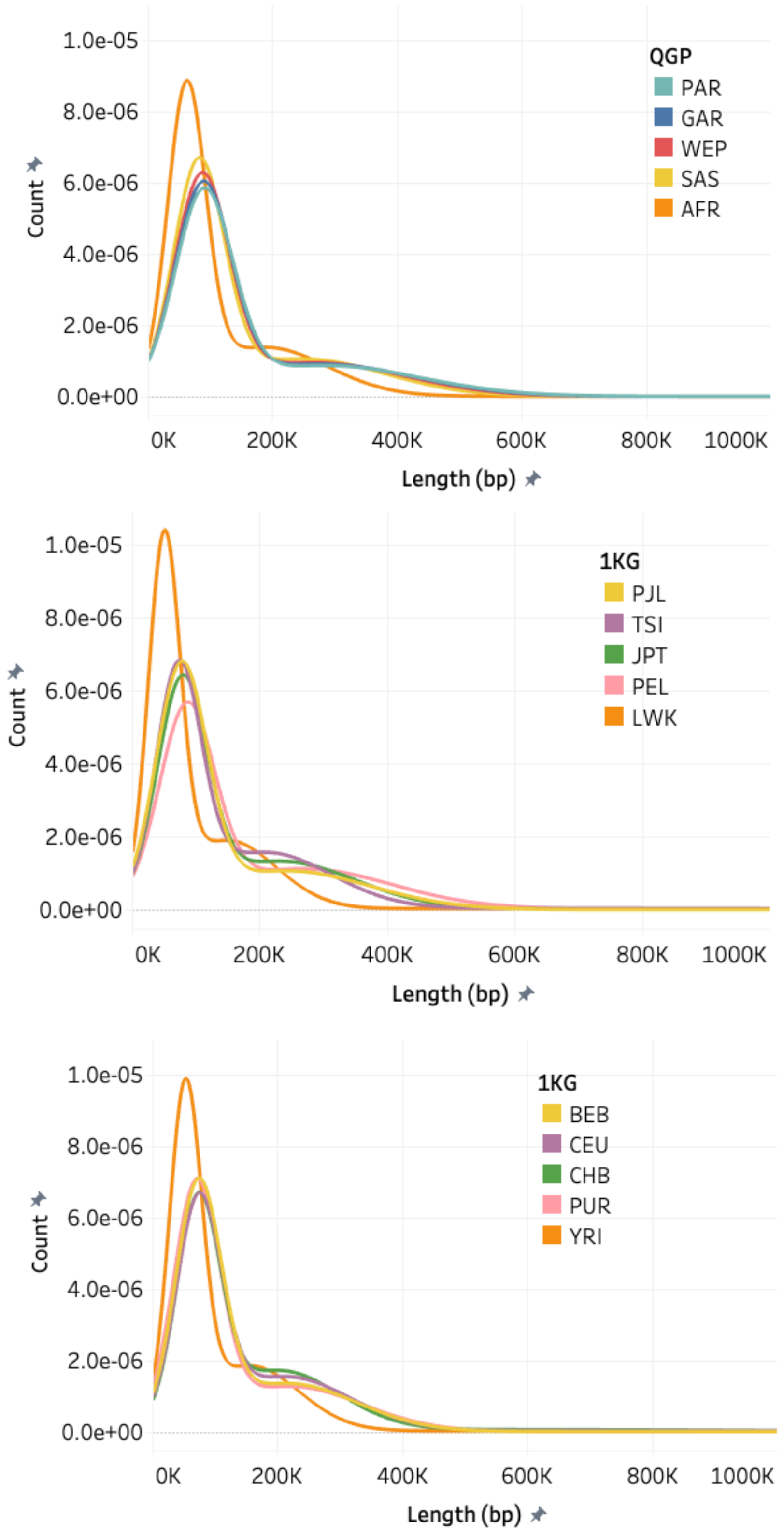

Supplementary Fig 17. Density plots showing abundance of ROH segments as a function of length, in QGP and 1KG populations.

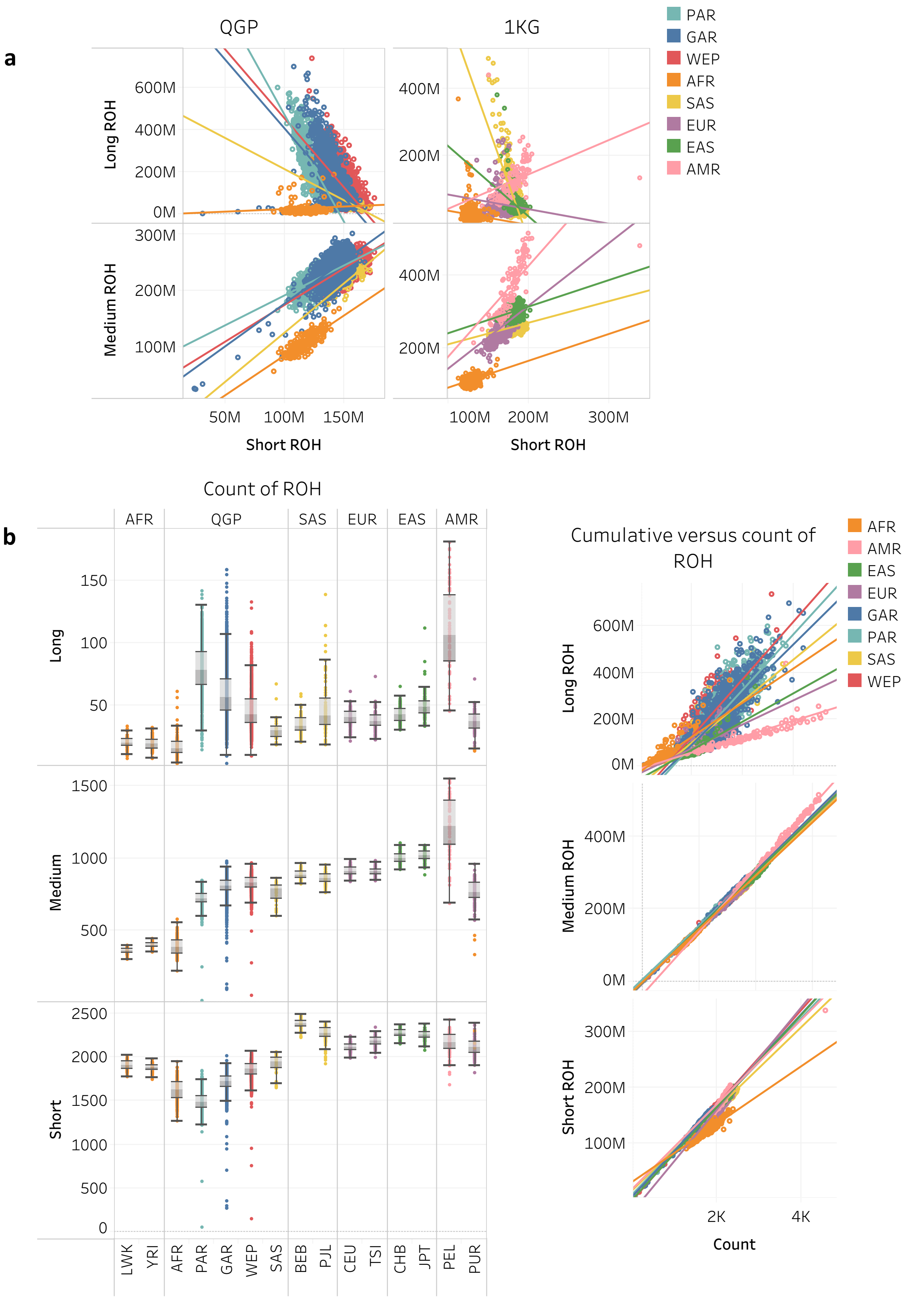

Supplementary figure 18. (a) Correlation of cumulative ROH between different ROH classes per population **(b)** Count of ROH segments in each size class per subject and population (left) and correlation with cumulative length (right). Numbers of samples used are the same as in Figure 5. Boxes indicate median and middle two quartiles of the data. Whiskers indicate data 1.5 times the interquartile range.

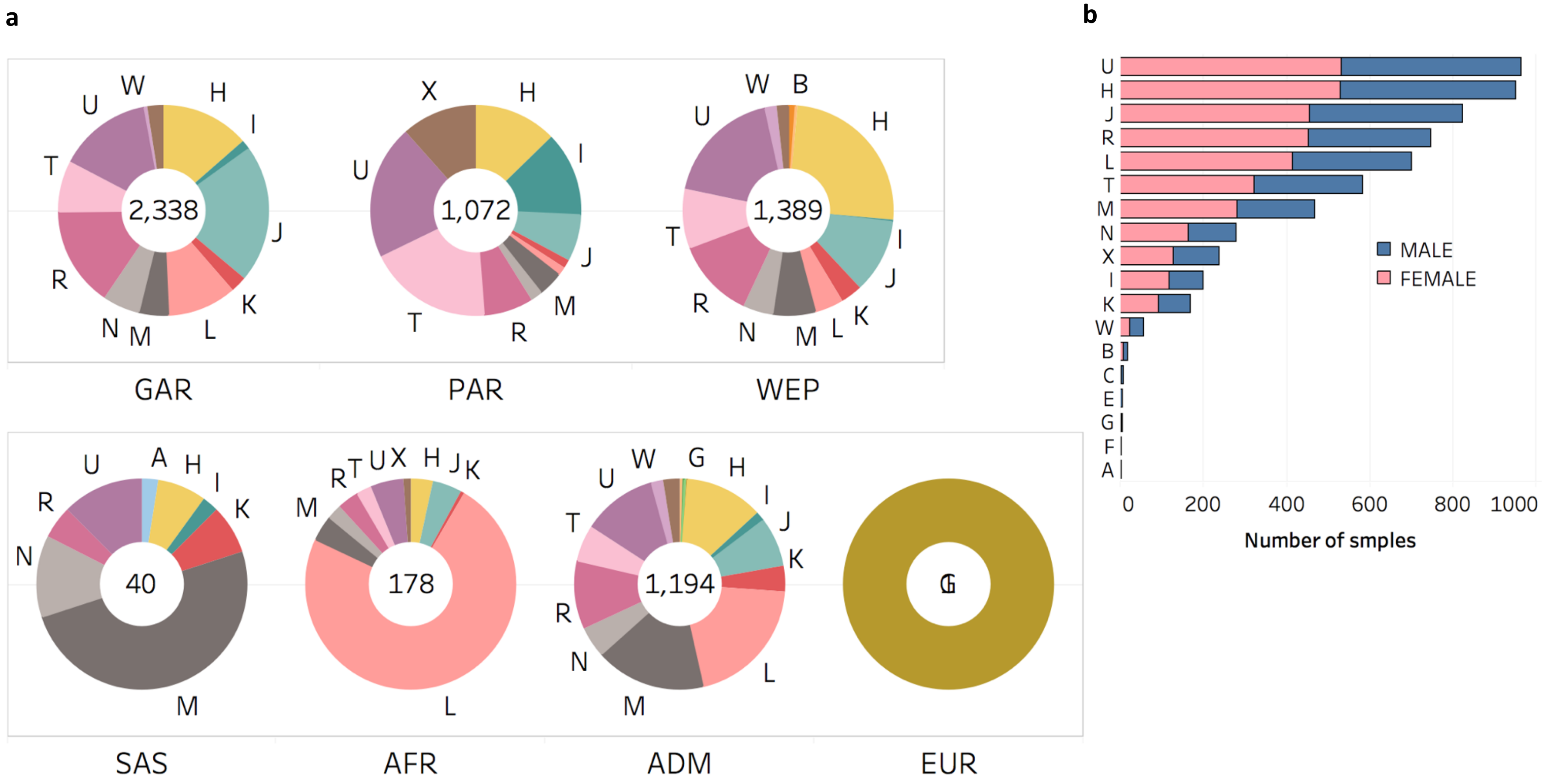

Supplementary Fig. 19. **mtDNA haplogroup assignment for QGP samples. (a)** Fractions of haplogroups per QGP sub-population. Each haplogroup is denoted by a distinct color. **(b)** Male and female composition in the QGP dataset.

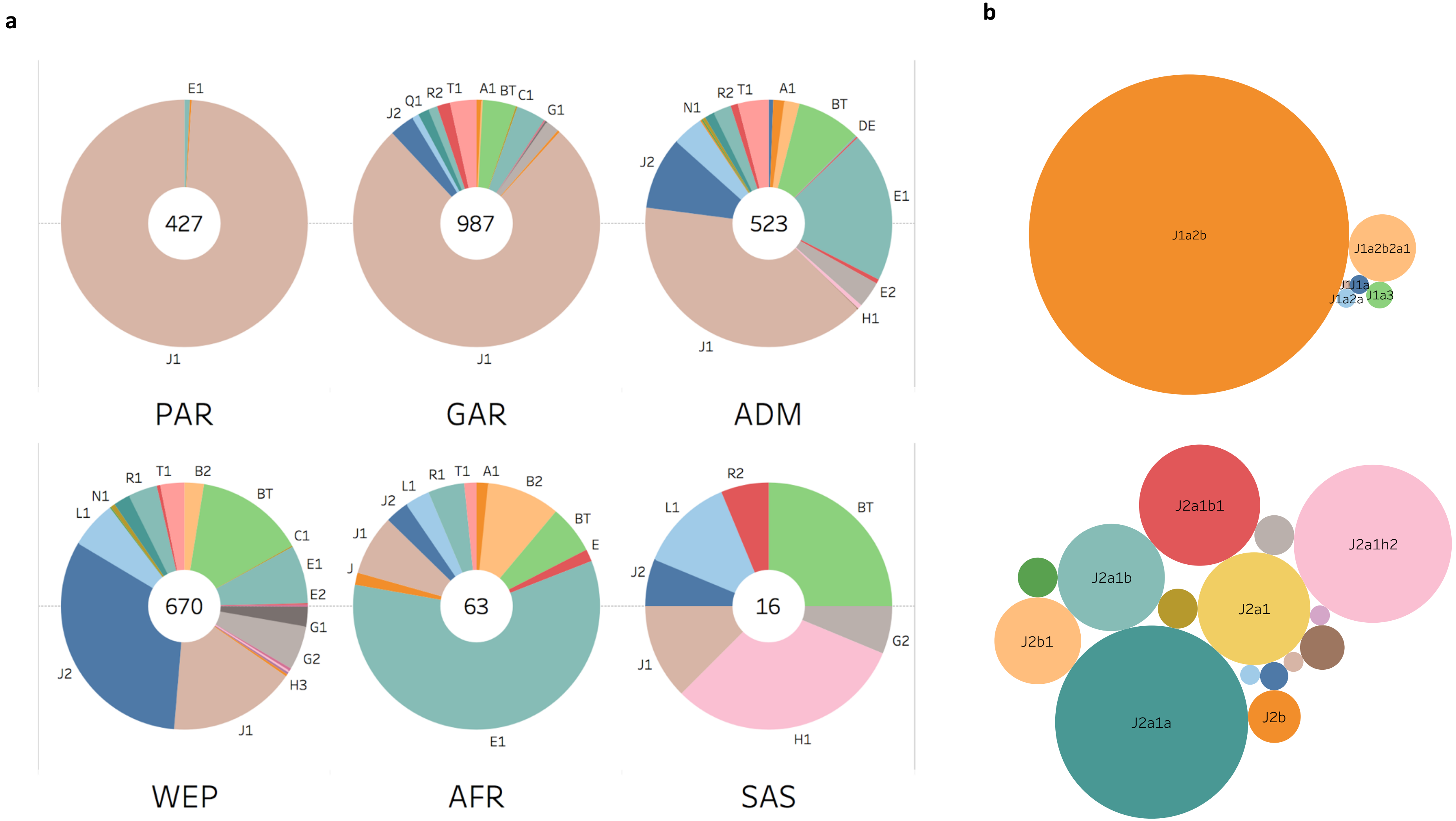

Supplementary Fig. 20. **Chromosome Y haplogroup assignment for QGP and 1KG samples. (a)** All haplogroups in QGP. **(b)** J1 and J2 in QGP. **(c)** all haplogroups in 1KG. **(d)** J1 and J2 in 1KG**.** Each haplogroup is denoted by a distinct color.

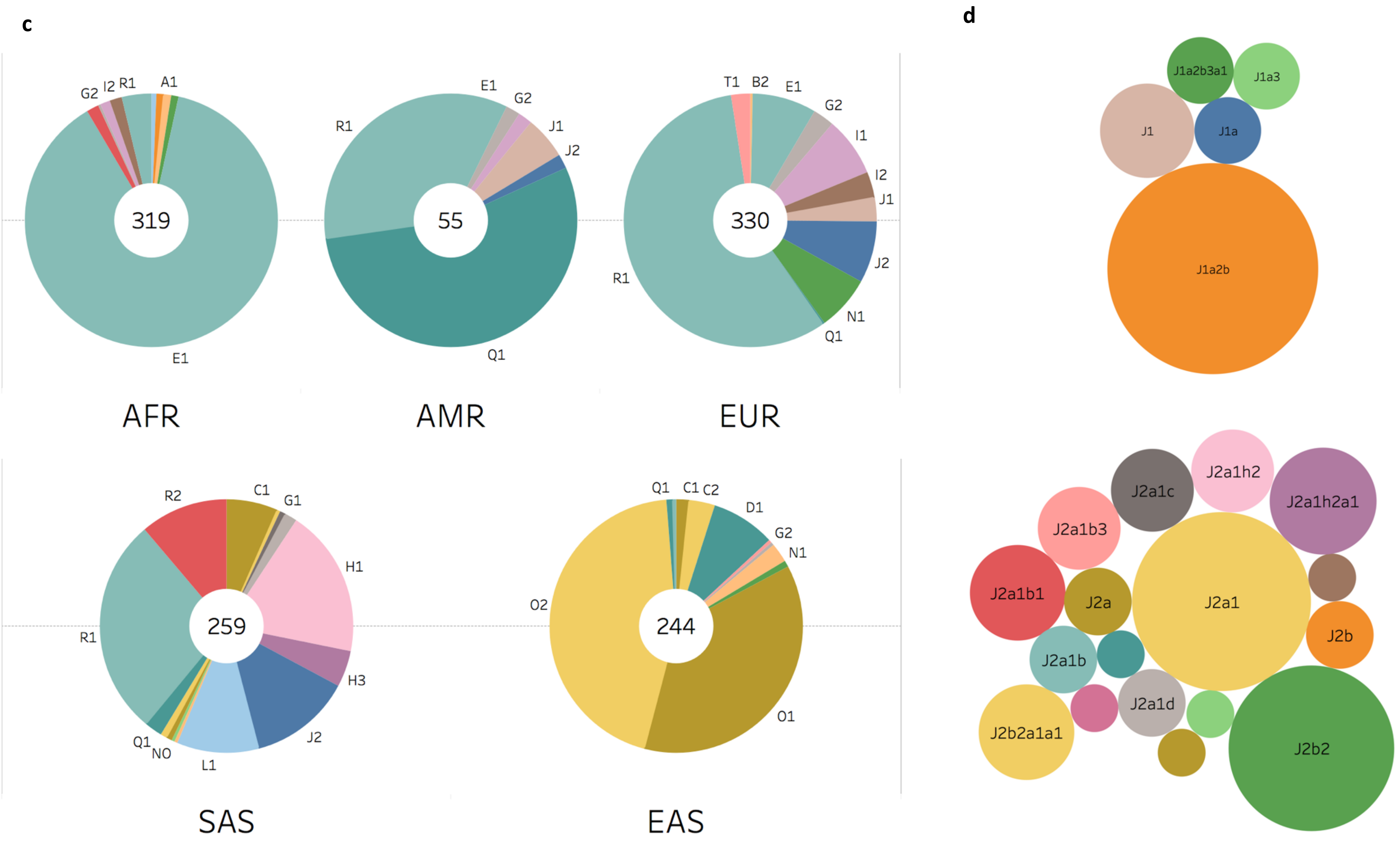

c
d
G2 I2 R1 A1
319
E1
AFR
R1 E1 G2 J1 J2
55
Q1
AMR
T1 B2 E1 G2 I1 I2 J1
330
R1 J2 N1 Q1
EUR
R2 C1 G1 H1
259
R1 H3 Q1 NO L1 J2
SAS
Q1 C1 C2 D1 G2 N1
O2
244
O1
EAS
J1a2b3a1
J1a3
J1
J1a
J1a2b
J2a1h2
J2a1c
J2a1h2a1
J2a1b3
J2a1b1
J2a
J2a1
J2b
J2a1b
J2a1d
J2b2a1a1
J2b2

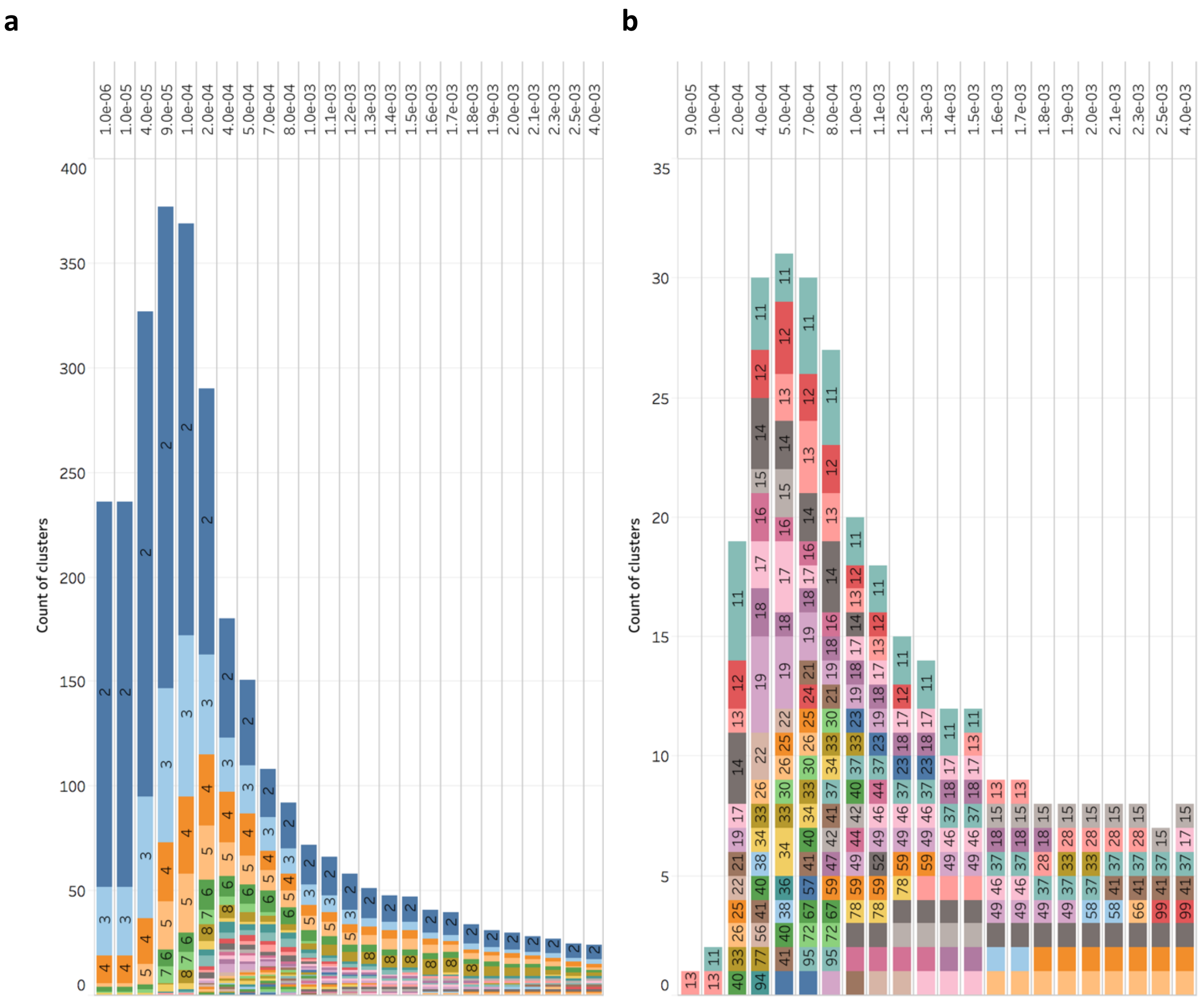

Supplementary Fig. 21. Number of clusters in the J1a2b tree as a function of cluster size and genetic distance cut-off. (a) For cluster size > 1 (Non-singletons) **(b)** For cluster size >10. Bars are colored, and labeled with cluster size. Singletons are excluded. Largest number of clusters with > 10 samples is observed at genetic distance cutoff of 5 x 10^{-4}.

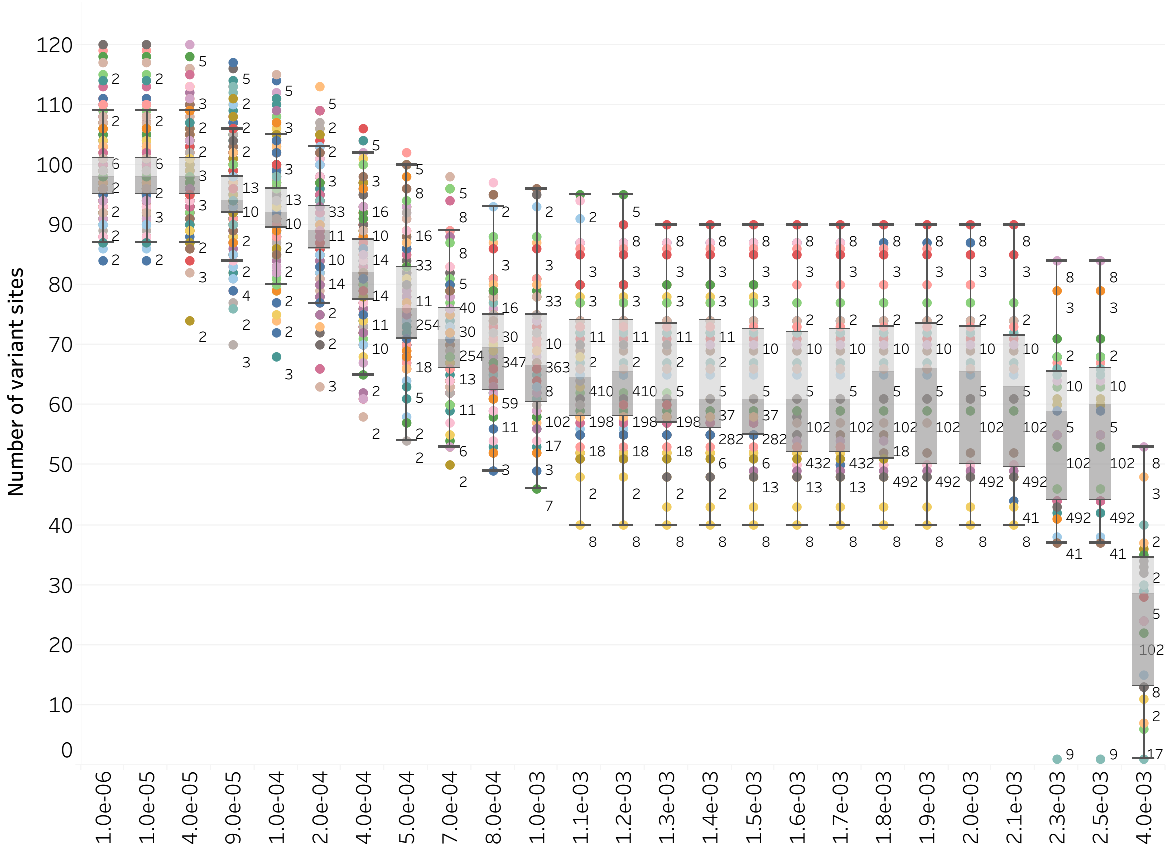

Supplementary Fig. 22. Distribution of haplogroup lengths for J1a2b samples as a function of genetic distance cut-off. Dots correspond to haplogroups and are labeled with the size of clusters in which the haplogroup occurs. Singletons are excluded. Boxes indicate median and middle two quartiles of the data. Whiskers indicate data 1.5 times the interquartile range. The underlying number of samples used is 1,426.

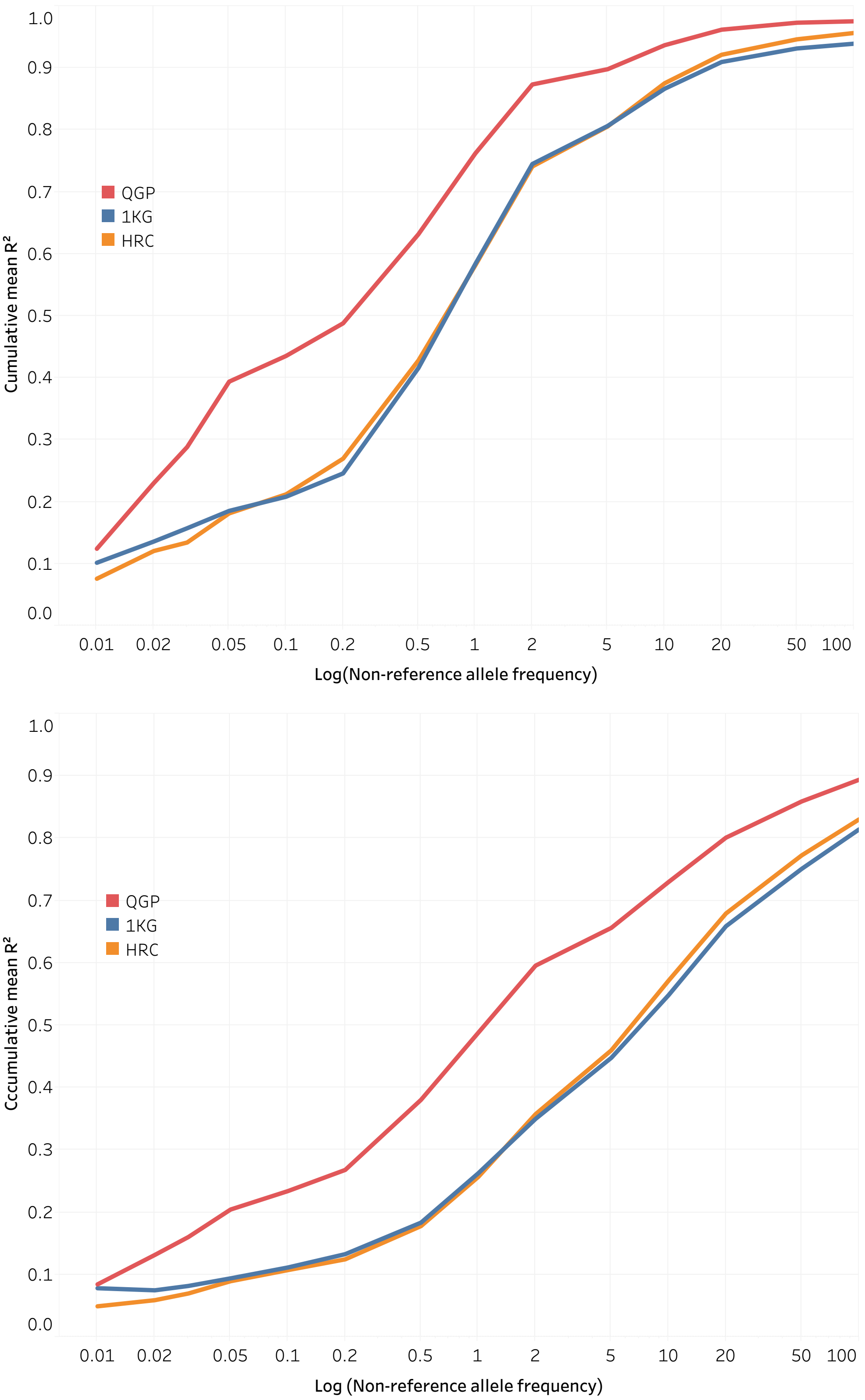

Supplementary Fig. 23. Imputation using QGP and other publicly available reference panels based on Omni5 and Core Exome pseudo arrays. Shown is non-reference allele frequency of imputed SNPs on a log scale against imputation accuracy measured by mean R^2 when imputing SNP genotypes into 105 independent Qatari samples. The results are based on genotypes on Omni 5 (Top) and Core Exome (bottom) pseudo arrays.
