# Supplementary material for: Thousands of Qatari genomes inform human migration history and improve imputation of Arab haplotypes
Source: Nat Commun. 2021 Oct 12;12:5929. doi: 10.1038/s41467-021-25287-y (PMC8511259; doi:10.1038/s41467-021-25287-y)
Supplement: Supplementary file 2 — Description of Additional Supplementary Files [file 41467_2021_25287_MOESM2_ESM.pdf]

Description of Additional Supplementary Files

File Name: Supplementary Data 1

Description: List of SNPs defining novel sun-haplogroups of the J1a2b Chr Y haplogroup. Sub-haplogroups were defined based on clusters with size > 10 generated by applying 5x10-4 genetic distance threshold on Maximum Likelihood tree with 90% bootstrapping.
