# Supplementary material for: Thousands of Qatari genomes inform human migration history and improve imputation of Arab haplotypes
Source: Nat Commun. 2021 Oct 12;12:5929. doi: 10.1038/s41467-021-25287-y (PMC8511259; doi:10.1038/s41467-021-25287-y)
Supplement: Supplementary file 4 — Reporting Summary [file 41467_2021_25287_MOESM4_ESM.pdf]

nature research

Corresponding author(s): Younes Mokrab
Khalid Fakhro

Last updated by author(s): Mar 4, 2021

Reporting Summary

Nature Research wishes to improve the reproducibility of the work that we publish. This form provides structure for consistency and transparency in reporting. For further information on Nature Research policies, see our Editorial Policies and the Editorial Policy Checklist.

Statistics

For all statistical analyses, confirm that the following items are present in the figure legend, table legend, main text, or Methods section.

n/a	Confirmed	
☐	☒	The exact sample size (n) for each experimental group/condition, given as a discrete number and unit of measurement
☐	☒	A statement on whether measurements were taken from distinct samples or whether the same sample was measured repeatedly
☐	☒	The statistical test(s) used AND whether they are one- or two-sided<br>*Only common tests should be described solely by name; describe more complex techniques in the Methods section.*
☒	☐	A description of all covariates tested
☐	☒	A description of any assumptions or corrections, such as tests of normality and adjustment for multiple comparisons
☐	☒	A full description of the statistical parameters including central tendency (e.g. means) or other basic estimates (e.g. regression coefficient) AND variation (e.g. standard deviation) or associated estimates of uncertainty (e.g. confidence intervals)
☐	☒	For null hypothesis testing, the test statistic (e.g. F, t, r) with confidence intervals, effect sizes, degrees of freedom and P value noted<br>*Give P values as exact values whenever suitable.*
☒	☐	For Bayesian analysis, information on the choice of priors and Markov chain Monte Carlo settings
☒	☐	For hierarchical and complex designs, identification of the appropriate level for tests and full reporting of outcomes
☒	☐	Estimates of effect sizes (e.g. Cohen's d, Pearson's r), indicating how they were calculated

Our web collection on statistics for biologists contains articles on many of the points above.

Software and code

Policy information about availability of computer code

Data collection	Illumina HiSeq X Software v3
Data analysis	BWA-MEM v0.7.7, SAMtools v1.2, Picard v1.117, Genome Analysis Toolkit (GATK) v3.4, vcftools v1.9, rtg-tools v3.8, PLINK v2.0, ADMIXTURE v1.3.0, TreeMix v1.3, iTOL 2019, ADMIXTOOLS v5.1, BCFTools v1.9, HaploGrep v2.1.20, yhaplo v1.0.18, RaxML v8.0, vcf2phylip 2.3, ClusterPicker v1.2.3, Variant Effect Predictor v84, Eagle v2.3.5, Minimac v3, measureAggregateRsquared, glactools v1.0.

For manuscripts utilizing custom algorithms or software that are central to the research but not yet described in published literature, software must be made available to editors and reviewers. We strongly encourage code deposition in a community repository (e.g. GitHub). See the Nature Research guidelines for submitting code & software for further information.

Data

Policy information about availability of data

All manuscripts must include a data availability statement. This statement should provide the following information, where applicable:

- Accession codes, unique identifiers, or web links for publicly available datasets
- A list of figures that have associated raw data
- A description of any restrictions on data availability

Access to data in this study will be made under controlled access through a dedicated portal by the QGP as per its policy. The informed consent given by the study participants does not cover posting of participant level phenotype and genotype data of Qatar Biobank (QBB)/Qatar Genome Project (QGP) in public databases. However, access to QBB/QGP data can be obtained through an established ISO-certified process by submitting a project request at https://www.qatarbiobank.org.qa/research/how-to-apply which is subject to approval by the QBB IRB committee.

Field-specific reporting

Please select the one below that is the best fit for your research. If you are not sure, read the appropriate sections before making your selection.

☒ Life sciences ☐ Behavioural & social sciences ☐ Ecological, evolutionary & environmental sciences

For a reference copy of the document with all sections, see nature.com/documents/nr-reporting-summary-flat.pdf

Life sciences study design

All studies must disclose on these points even when the disclosure is negative.

Sample size	We used the dataset provided by Qatar Genome Project phase 1 consisting of whole genome of 6,218 predominantly healthy individuals recruited from the general population at Qatar Biobank. This is the data that was made available to us as end users and we don't have control to do sample size caclulation. However, this dataset is the largest ever published for a Middle Eastern population to date.
Data exclusions	Two samples were removed during the QC stage due to high level of missingness as mentioned in the manuscript. See Methods section for details.
Replication	We included various measures in order to obtain statistical confidence of our measurements, namely applying various clustering parameters k and 5 times cross validation (Admixture analysis), using different sets of SNPs based on R2 correlation thresholds for PCA analysis (0.2, 0.5 and 0.8); calculation of Z-score and standard error (F3 statistics and D-statistics); estimation based on multiple chromosomes (n=22) and multiple samples per population (n=86-201) (Effective population size and divergence history analysis); Calculation of ROH for all subjects of study populations and using two representative subpopulation per 1KG dataset (ROH analysis); Calculation using all individuals in the dataset not just a subset (Y and Mt haplogorups); Used Maximum likelihood method for the phylogenetic inference and applying various genetics distance cutoffs for defining clusters (Chr Y J1a2b clustering). Using independent and diverse set of test samples (n=108) for testing imputation accuracy (Imputation analysis).
Randomization	The randomization is not relevant in this study. This is a population-based cohort study.
Blinding	The blinding is not relevant in this study. This is a longitudinal population-based cohort study.

Reporting for specific materials, systems and methods

We require information from authors about some types of materials, experimental systems and methods used in many studies. Here, indicate whether each material, system or method listed is relevant to your study. If you are not sure if a list item applies to your research, read the appropriate section before selecting a response.

Materials & experimental systems

n/a	Involved in the study
☒	☐ Antibodies
☒	☐ Eukaryotic cell lines
☒	☐ Palaeontology and archaeology
☒	☐ Animals and other organisms
☐	☒ Human research participants
☒	☐ Clinical data
☒	☐ Dual use research of concern

Methods

n/a	Involved in the study
☒	☐ ChIP-seq
☒	☐ Flow cytometry
☒	☐ MRI-based neuroimaging

Human research participants

Policy information about studies involving human research participants

Population characteristics	Predominantly healthy individuals recruited from the general population at Qatar Biobank, aged 18 or over and nationals. 56.7 % of the participants were females whereas 43.3% were males.
Recruitment	Recruited as a sample population cohort at Qatar Biobank.
Ethics oversight	All QBB participants signed an Informed Consent Form prior to their participation. QBB study protocol ethical approval was obtained from Hamad Medical Corporation Ethical Committee in 2011 and continued with QBB Institutional Review Board (IRB) from 2017 onwards.

Note that full information on the approval of the study protocol must also be provided in the manuscript.
